# Heat Shock Strengthens the Protective Potential of MSCs in Liver Injury by Promoting EV Release Through Upregulated Autophagosome Formation

**DOI:** 10.1002/jev2.70084

**Published:** 2025-05-06

**Authors:** Tingting Wang, Yihang Gong, Huizhu Lin, Xuejiao Li, Jinliang Liang, Xiaofeng Yuan, Cuiping Li, Zhongying Hu, Haitian Chen, Jiaqi Xiao, Jiebin Zhang, Yasong Liu, Xijing Yan, Chenhao Jiang, Jia Yao, Qi Zhang, Rong Li, Jun Zheng

**Affiliations:** ^1^ Organ Transplantation Research Center of Guangdong Province, Department of Hepatic Surgery and Liver Transplantation Center of the Third Affiliated Hospital of Sun Yat‐sen University Guangdong Province Engineering Laboratory for Transplantation Medicine Guangzhou China; ^2^ Guangdong Provincial Key Laboratory of Liver Disease Research Third Affiliated Hospital of Sun Yat‐sen University Guangzhou China; ^3^ Biological Treatment Center The Third Affiliated Hospital of Sun Yat‐sen University Guangzhou China; ^4^ Department of General Intensive Care Unit Lingnan Hospital, The Third Affiliated Hospital of Sun Yat‐sen University Guangzhou China; ^5^ Department of Breast and Thyroid Surgery Lingnan Hospital, The Third Affiliated Hospital of Sun Yat‐sen University Guangzhou China; ^6^ Biotherapy Centre & Cell‐gene Therapy Translational Medicine Research Centre The Third Affiliated Hospital, Sun Yat‐sen University Guangzhou China

**Keywords:** extracellular vesicles release, heat shock, mesenchymal stem cells, succinylation modification, TRPV2

## Abstract

Mesenchymal stem cell‐derived extracellular vesicles (MSC‐EVs) show powerful potential in the treatment of multiple diseases. However, the low yield of MSC‐EVs severely restricts their clinical application. Here, heat shock (HS), a moderate external stimulus, can enhance EVs release of MSCs by upregulating autophagosome formation. Mechanistically, HS elevates TRPV2 expression to induce Ca^2+^ influx and then promotes the activity of two succinylases, SUCLG2 and OXCT1, followed by increasing the succinylation of YWHAZ (a 14‐3‐3 protein) at lysine 11 (K11). Acting as an adaptor protein, YWHAZ's succinylation at K11 inhibits its degradation, reinforcing YWHAZ‐ULK1 binding, which upregulates ULK1 S555 phosphorylation to promote autophagosome formation and enhance EV release of MSCs. Additionally, the improved therapeutic efficacy of HS‐treated MSCs via EV release has been shown in two liver injury models—hepatic ischemia/reperfusion injury (HIRI) and acetaminophen‐induced liver injury. These findings proved that HS, an easily implementable and cost‐effective method, can be used to elevate MSC‐EV yield in mass production.

## 1 Introduction

Mesenchymal stem/stromal cells (MSCs) are extensively derived from umbilical cords (UCs), bone marrow, dental pulp, adipose tissue, and placental tissue. Numerous studies have demonstrated their therapeutic effects in inflammatory disorders and tissue repair and regeneration (Lan et al. 2021). To date, over 1500 MSC clinical trials involving 11,000 patients have been registered on ClinicalTrials.gov. Previously, we demonstrated the roles of MSCs in attenuating liver diseases and acute lung injury (ALI) (Zheng, Chen, et al. 2020; Zheng et al. 2019; Lv et al. 2020) and conducted clinical studies to reveal MSCs could prevent severe postoperative complications after liver transplantation and treat acute respiratory distress syndrome (ARDS) (Lv et al. 2020; Zhang et al. 2021; Zhang et al. 2017).

MSC‐derived extracellular vesicles (MSC‐EVs), ranging from 30 to 300 nm in size with bilipid membranes, have been reported to carry functional contents and exhibit similar therapeutic potentials as the parent MSCs. A current research hotspot is the evaluation of the feasibility of using MSC‐EVs to replace MSCs in medical applications, given their high biosafety and unique advantages, including low rates of tumorigenicity and pulmonary embolism (Yao et al. 2019; Zheng, Lu, et al. 2020). Our previous studies also showed MSCs alleviate hepatic ischemia/reperfusion injury (HIRI) (Yao et al. 2019; Zheng, Lu, et al. 2020; Lu et al. 2022) and promote liver regeneration in aged models via MSC‐EVs (J. Zhang et al. 2023). Given the promising effects of MSC‐EVs, clinical trials assessing their safety and efficacy for various diseases are underway. However, the low yield of EVs from MSCs remains a significant limitation for their clinical application, necessitating the exploration of viable methods to enhance EV production of MSCs (Neupane et al. 2023).

It is widely accepted that moderate harmful stress can enhance the functions and maintain the status of stem cells (Choudhery et al. 2015). Among these, heat shock (HS), a short‐term hyperthermia treatment (38°C–42°C), can promote the synthesis of self‐protective proteins and stimulate cellular functions. Previous studies have shown that HS enhances the osteogenic potential of MSCs (Abd‐El‐Aziz et al. 2022; Chen et al. 2013). Additionally, HS improves MSC viability by upregulating the expression of HS proteins 70 and 90 (HSP70 and HSP90) (Q. Wang et al. 2019). Qiao demonstrated that HS not only enhances MSC survival but also improves their hepatoprotective roles (Qiao 2015). Our recent study also revealed that HS can enhance the pulmonary protective potential of MSCs by upregulating HSP70 to inhibit the NLRP3 inflammasome formation in macrophages (Lv et al. 2021). However, whether HS can increase the MSCs‐EVs yield and the underlying mechanisms remain unclear.

In this study, we demonstrated that HS can increase EV release from MSCs via upregulating the transient receptor potential vanilloid 2 (TRPV2) expression. TRPV2, a calcium channel, can induce calcium influx to enhance the activity of two succinylases (SUCLG2 and OXCT1), which increases YWHAZ succinylation and subsequently stimulates autophagosome formation. Additionally, we established two liver injury models—HIRI and acetaminophen (APAP)‐induced liver injury—to confirm that HS enhances the hepatoprotective potential of MSCs by increasing EV yield. Our work could be leveraged to enhance the yield of EVs derived from MSCs and promote the clinical translational application of MSC‐EVs in the future.

## 2 Results

### 2.1 HS Treatment Promotes EV Release From MSCs

**FIGURE 1:**
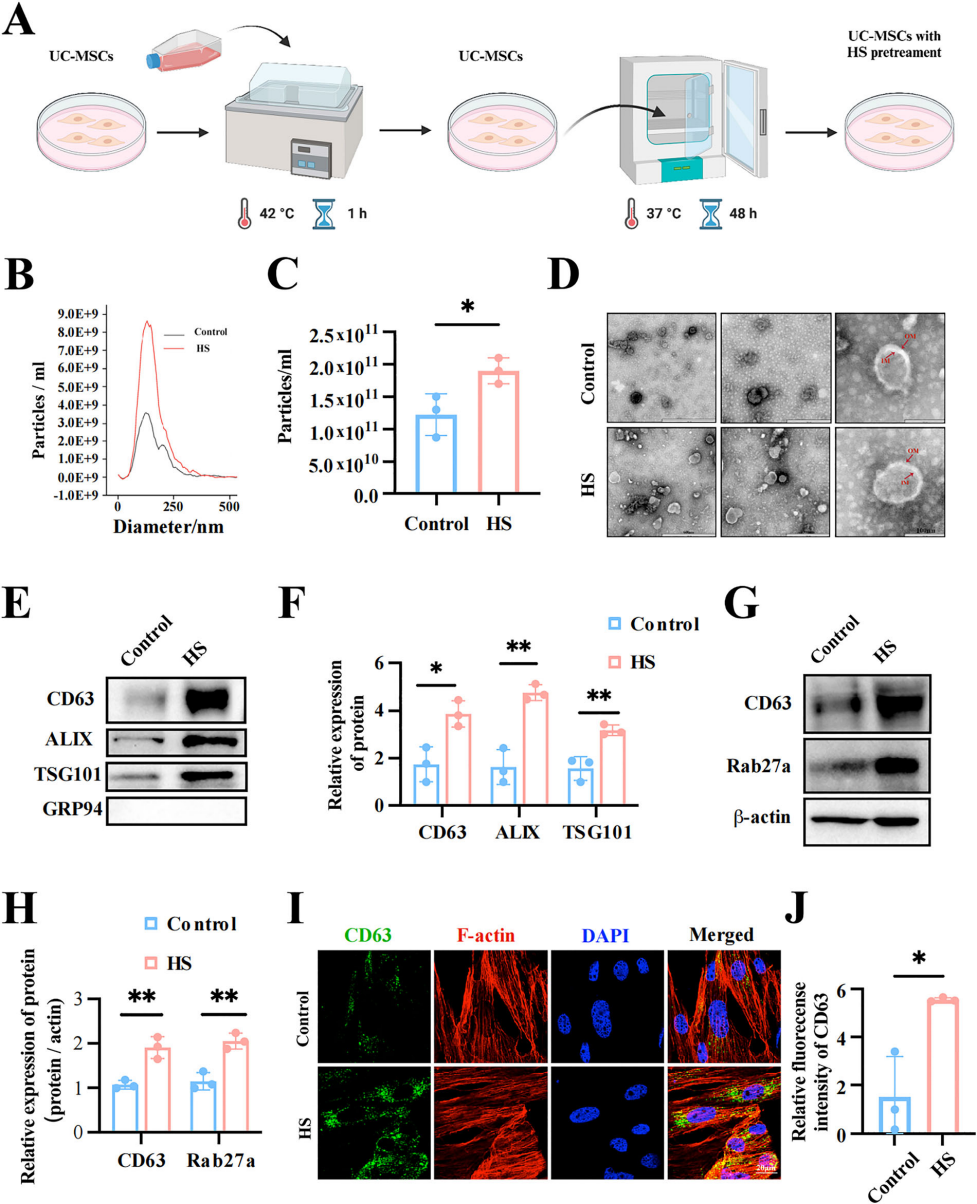
HS treatment promotes EVs release of MSCs. (A) Schematic representation of the HS treatment. (B, C) The isolated EVs of indicated MSCs were examined by NTA and the particle concentration was analysed. (D) TEM showed the morphology of EVs (scale bar: 600 nm, 300 nm, and 100 nm). (E, F) EVs from both normal group and HS treatment group were assessed by western blotting and the relative protein levels were calculated. (G, H) Cell lysates from MSCs treated with or without HS were assessed by western blotting and the relative protein levels were calculated. (I, J) Represent images of CD63 (green), together with F‐actin (red) were detected by confocal microscopy (scale bar: 20 µm) and the relative fluorescence intensity was analysed. All experiments of EVs collection were subjected to a standardized 48‐hour incubation protocol. Data are shown as the mean ± SD (*n*  =  3). *p < 0.05.

To investigate the potential of HS treatment in EV yield, we first isolated MSCs from UCs. These cells exhibited a fibroblast‐like morphology, displayed osteocytes and adipocytes differentiation potential as well as positively expressed CD73, CD90, and CD105, and negatively expressed CD11b, CD19, CD45, CD34, and HLA‐DR (Figure S1A–C). Following a 1‐h HS treatment, cells were cultured for 48 h under standard conditions (Figure 1A). MSC‐related characteristics and cell viability were not influenced by HS treatment (Figure S1A–E).

Then, we isolated EVs from the culture medium of control or HS‐treated groups to evaluate the influence of HS treatment on EV yield. Nanosight tracking analysis (NTA) and transmission electron microscopy (TEM) assays showed that HS can indeed significantly increase EVs release from an equal number of MSCs compared to the control group (Figure 1B,D). Western blotting analysis of EV‐related markers, including CD63, Alix, TSG1t01, and contaminating marker GRP94 (a negative control), further proved that HS could increase EV secretion (Figure 1E,F). Apart from the detection of EVs in cell culture medium, the number of intraluminal vesicles (ILVs) and their release capacity of indicated MSCs were also evaluated via the expression of CD63 and Rab27a (a member of the Rab GTPases family, which regulates exosome secretion by controlling the fusion of multivesicular bodies (MVBs) with the plasma membrane). As shown in Figure 1G–J, HS treatment significantly upregulated the protein levels of CD63 and Rab27a and induced higher levels of CD63‐stained vesicular structures in MSCs.

### 2.2 Autophagosome Formation Promotes EV Release From HS‐Treated MSCs

Autophagy, an important intracellular degradation system, can respond to stress by delivering potentially harmful or superfluous constituents to lysosomes for degradation. Previous studies demonstrated autophagy can promote EV release via the fusion of autophagosomes and multivesicular bodies (MVB) (Dias et al. 2016). Lei et al. found hypoxia stimulated autophagy in cancer‐associated fibroblasts to promote EV release (Xi et al. 2021). Additionally, HS can also stimulate autophagy (Dokladny et al. 2015). Therefore, we speculated HS treatment may promote EV release by increasing autophagosome formation.

**FIGURE 2:**
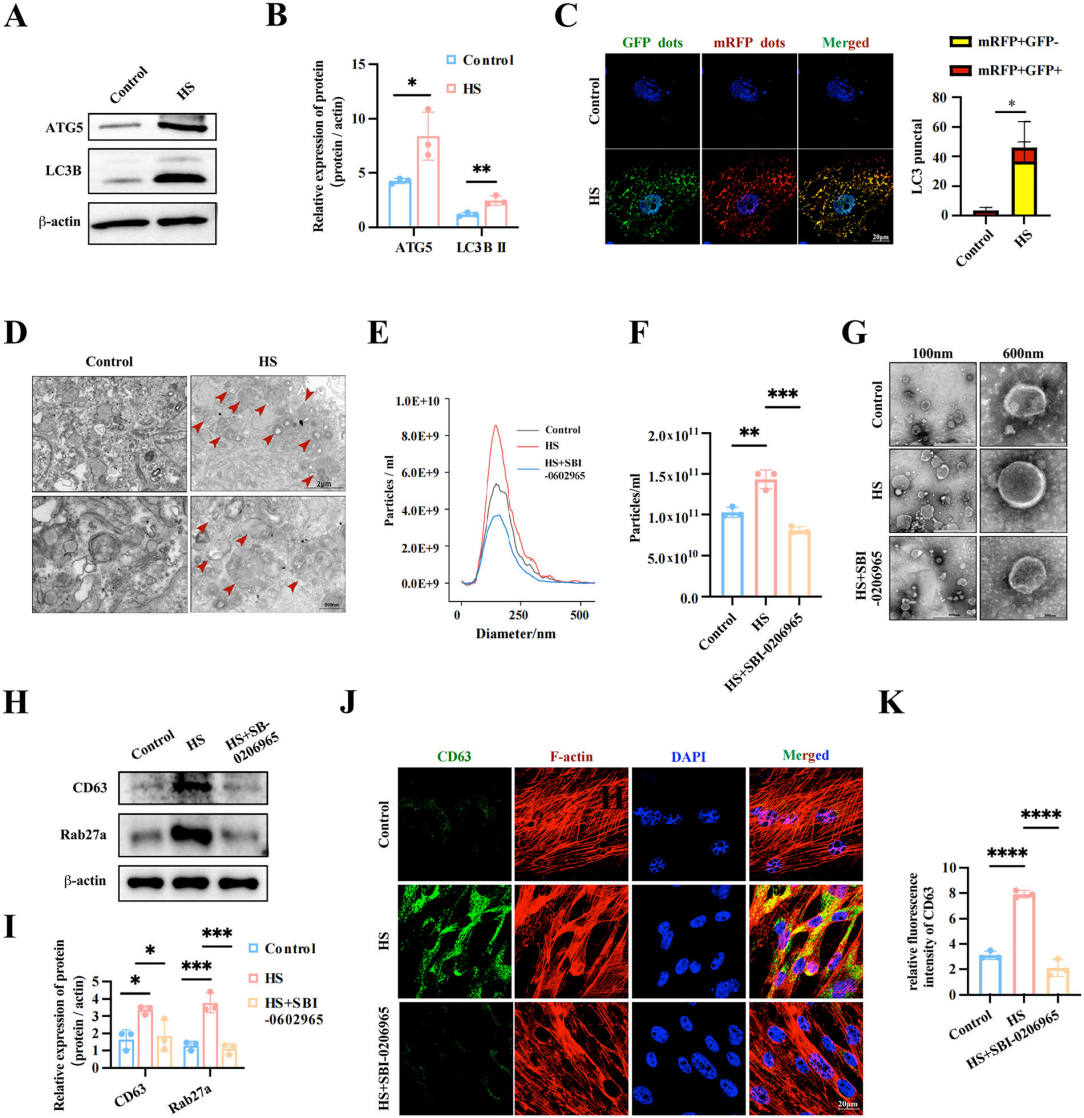
Autophagosome formation promotes EVs release from MSCs treating with HS. (A, B) western blotting analysis of autophagy‐related proteins in MSCs treated with or without HS treatment and the relative protein levels were calculated. (C) MSCs were transfected with GFP‐mRFP‐LC3 AAVs and treated with or without HS. The autophagosomes were shown as yellow puncta with both mRFP (red) and GFP (green) labels, autolysosomes were shown as red only puncta because after fusion with lysosomes, GFP loses its fluorescence in acidic PH. DAPI (blue) was used to stain nuclei (scale bar, 20 µm). (D) TEM of MSCs of each group as indicated. The arrows indicate autophagic vacuoles. (E, F) The isolated EVs were examined by NTA and the particle concentration was analysed. (G) TEM showed the morphology of EVs (scale bar: 600 nm, 300 nm and 100 nm). (H, I) Cell lysates from MSCs treated with HS or SBI‐0206965 combined with HS were assessed by western blotting and the relative protein levels were calculated. (J, K) Represent images of CD63 (green), together with F‐actin (red) of indicated MSCs were detected by Confocal microscopy (scale bar: 20 µm) and the relative fluorescence intensity was analysed. All experiments of EVs collection were subjected to a standardized 48‐h incubation protocol. Data are shown as the mean ± SD (*n*  =  3). *p < 0.05, **p < 0.01, ***p < 0.001, ****p < 0.001.

Based on the detection of autophagy‐related proteins (Atg5 and the LC3II/I ratio), we found HS treatment indeed upregulated autophagy in MSCs (Figure 2A,B and Figure S2A,B), which was confirmed with immunofluorescence staining and TEM analyses (Figure 2C,D).

Next, we used rapamycin, an autophagy agonist, and SBI‐0206965, an autophagy antagonist, to further validate this phenomenon. As expected, rapamycin served as a positive control, while SBI‐0206965 suppressed the expression of autophagy‐related proteins and autophagosome formation induced by HS treatment (Figure S2C–E). Subsequently, we detected whether autophagosome formation influenced HS treatment‐induced EV release in MSCs. As shown in Figure S2F,G, rapamycin significantly increased EV release. While SBI‐0206965 strongly reversed the effect of HS treatment on the EV release (Figure 2E–G). These effects were confirmed with the alterations on the protein levels of CD63, Alix, and TSG101 in the total EVs (Figure S2H,I). Moreover, we also proved that regulating autophagosome formation could significantly affect the levels of ILVs and their release capacity of indicated MSCs (Figure 2H–K and Figure S2J–M).

### 2.3 HS Treatment Activates Autophagy to Stimulate EV Release From MSCs by Upregulating TRPV2 Expression

**FIGURE 3:**
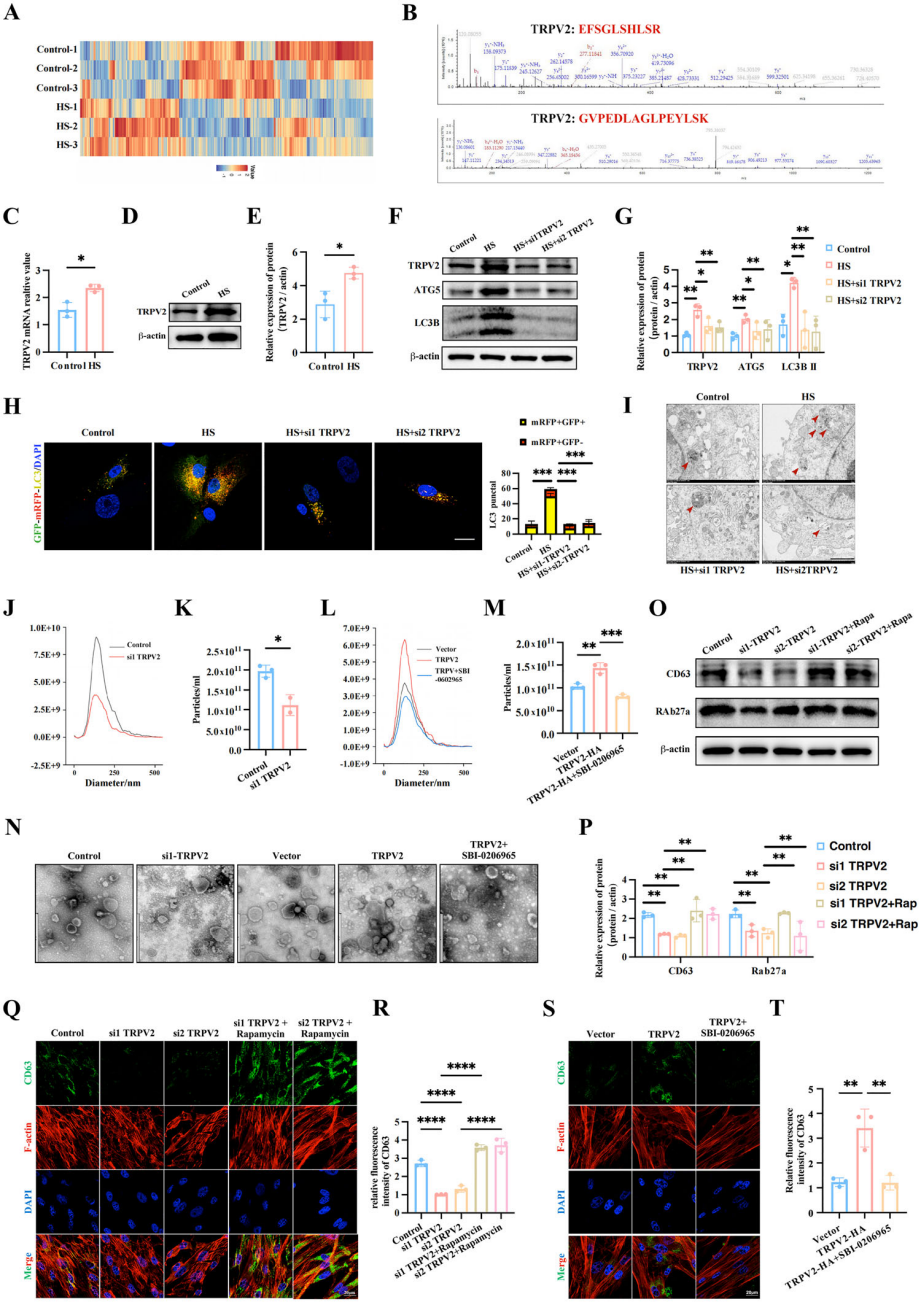
**HS treatment activates autophagy to stimulate EVs release of MSCs by upregulating TRPV2 expression**. (A) Heatmap showing differentially expressed proteins in the different groups. (B)The ion chromatograms of TRPV2. (C) Relative mRNA expression levels of TRPV2 in MSCs treated with HS and control were determined using RT‐qPCR; GAPDH served as an internal control. (D, E) The expression of TRPV2 in both normal group and HS treatment group were assessed by western blotting and the relative protein levels were calculated. (F, G) The effects of TRPV2 on the expression of autophagy‐related proteins were assessed by western blotting and the relative protein levels were calculated. (H) Image analysis of GFP/mRFP puncta in MSCs of each group as indicated. Scale bar: 20 µm. (I) TEM of MSCs of each group as indicated. The arrows indicate autophagic vacuoles. Scale bar: 2 µm. (J–M) The isolated EVs from indicated MSCs groups were examined by NTA and the related particle concentration was analysed. (N) TEM showed the morphology of EVs (scale bar: 600 nm). (O, P) Western blotting showed the expression of CD63 and Rab27a of each group as indicated and the relative protein levels were calculated. (Q–T) Immunofluorescence staining images of DAPI (blue), CD63 (green) and F‐actin (red) in each group and the relative fluorescence intensity was analysed (Scale bar, 20 µm). All experiments of EVs collection were subjected to a standardized 48‐h incubation protocol. Data are shown as the mean ± SD (*n*  =  3). *p < 0.05, **p < 0.01, ***p < 0.001.

To explore the underlying mechanism by which HS upregulated autophagy, LC‐MS/MS analysis was conducted to compare protein differences between the normal and HS‐treated groups. Principal component analysis (PCA) indicated distinct group separations (Figure S3A). Heatmaps and volcano plot displayed the altered proteins between these two groups (Figure 3A and Figure S3B). Among them, TRPV2 was notably upregulated in the HS‐treated group (Figure 3B). TRPV2, a member of the TRPV family, is a thermal sensation protein widely distributed in multiple cells and involved in regulating calcium influx. Western blotting and RT‐qPCR assays confirmed the upregulation of TRPV2 mediated by HS treatment (Figure 3C–E and Figure S3C,D). Additionally, HS‐induced TRPV2 was primarily localized on the membrane of MSCs (Figure S3E).

As TRPV2 is a calcium‐permeable channel, immunofluorescence staining confirmed that HS significantly induced Ca^2+^ influx into MSCs (Figure S3F,G). To determine whether TRPV2 affects HS‐induced autophagosome formation, MSCs were transfected with siTRPV2 or a TRPV2 overexpression plasmid (Figure S3H,I). Figure 3F–I and Figure S3J–M showed that suppressing TRPV2 not only reduced intracellular Ca^2+^ concentration but also downregulated the HS‐induced expression of autophagy‐related proteins and autophagosome formation, and vice versa.

To further investigate whether TRPV2 stimulates autophagy and subsequently promotes EV release in HS‐treated MSCs, we co‐treated MSCs with siTRPV2 or the TRPV2 overexpression plasmid along with rapamycin or SBI‐0206965. EVs isolation from each group demonstrated that TRPV2 upregulated EV release, and blocking autophagy reversed this effect (Figure 3J–N).

Additionally, we detected the effect of TRPV2 on ILVs’ number and their release capacity of MSCs. Compared to the normal group, TRPV2 knockdown significantly inhibited the expression of CD63 and Rab27a, while rapamycin reversed this effect (Figure 3O,P and Figure S3N–Q). Conversely, TRPV2 overexpression upregulated EV release‐related proteins, but co‐treatment with SBI‐0206965 reduced their levels (Figure 3O,P and Figure S3N–Q). Similar trends were observed in the results of CD63 immunofluorescence staining (Figure 3Q–T). In summary, these findings suggest that HS treatment enhances autophagy to increase EV secretion from MSCs, in part by upregulating TRPV2 expression.

### 2.4 Global Landscape of Succinylation in MSCs Treated With HS

**FIGURE 4:**
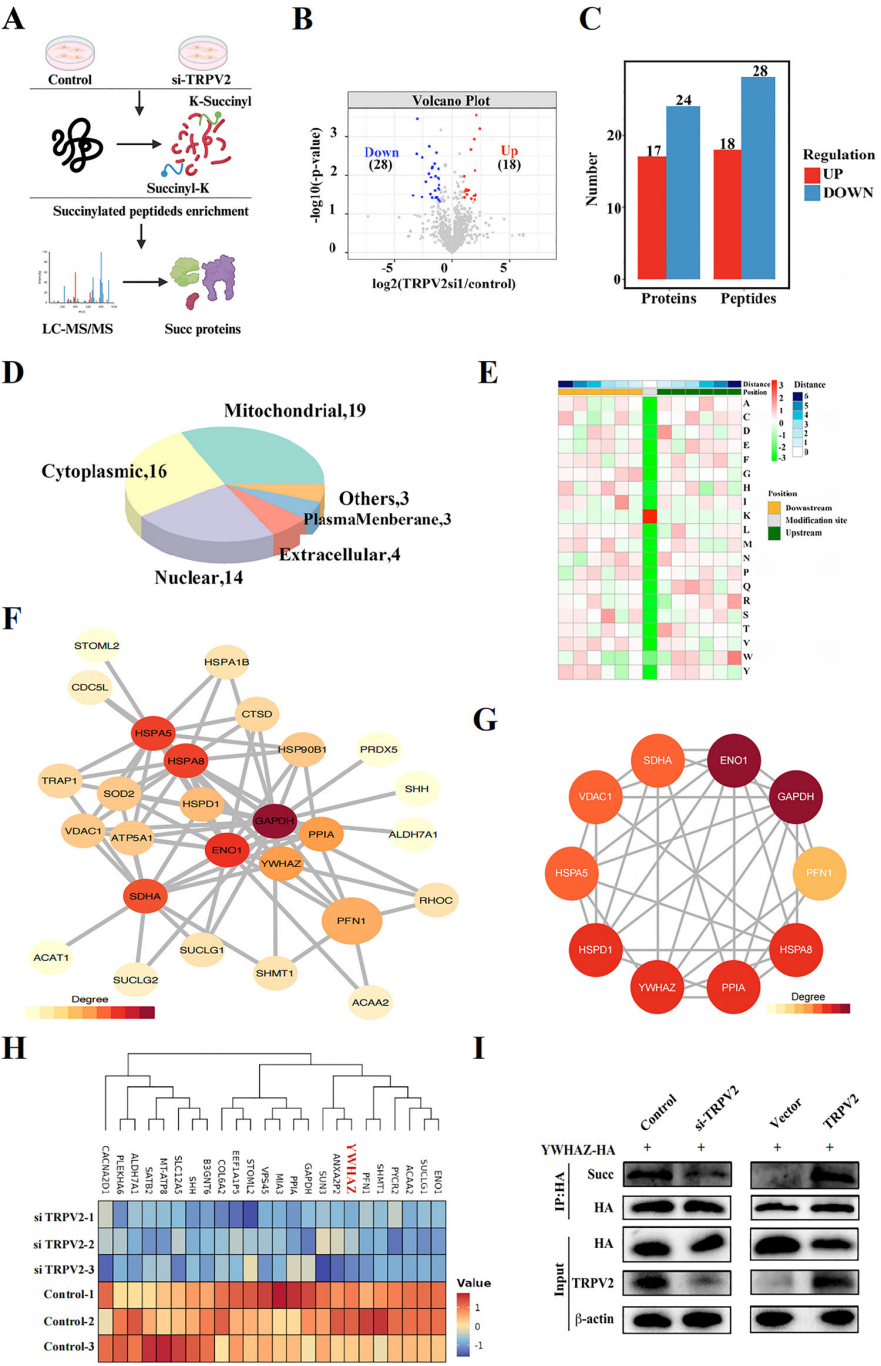
**Global Landscape of Succinylation in MSCs Treated with HS**. (A) Scheme of the lysine succinylome in MSCs with or without HS treatment. (B) Volcano plot showing the significant differentially expressed lysine succinylation (Succ) peptides between control and HS treated Group. (C) Bar charts showing the differentially expressed Succ proteins (DESPs) and differentially expressed Succ peptides identified in control and HS treated MSCs. (D) Subcellular distribution of DESPs in control and HS treated MSCs. (E) Heatmap indicating the enrichment (yellow) and depletion (green) of amino acids at each position flanking the Succ sites. (F) PPI network showing the relationships among the DESPs. (G) Top 10 hub proteins in the PPI network. (H) Heatmap showing the DESPs identified by succinylome analysis between two indicated groups. (I) Succ modification of YWHAZ in MSCs was regulated by TRPV2.

Previous studies showed that external stress, such as HS, can induce transient changes in the modification of intracellular proteins (Cheng et al. 2023; Sheng et al. 2024). To explore the underlying mechanism of TRPV2‐induced autophagy in MSCs during HS treatment, we compared 5 common modifications (acetylation, SUMOylation, malonylation, β‐hydroxybutyrylation, and succinylation) between the indicated group. A significant increase in succinylation was observed when MSCs received HS treatment, while silencing TRPV2 significantly decreased this modification (Figure S4A,B). Other modifications showed no significant difference. To reveal TRPV2‐related succinylation in MSCs, we used succinylation pan‐antibody‐conjugated beads to enrich modified peptides, followed by LC‐MS/MS analysis (Figure 4A).

We found 18 succinylated peptides specifically upregulated and 28 succinylated peptides downregulated in MSCs transfected with siTRPV2 (Figure 4B). All these succinylated peptides were identified in 41 proteins (Figure 4C). The pie chart showed the intracellular distribution of these differentially expressed succinylated proteins (Figure 4D). Analysis of the amino acid sequences surrounding succinylation sites revealed that the −1 and +1 positions of these sites were negatively charged (Figure 4E). All differentially expressed succinylated sites were defined by four subgroups according to their succinylated levels’ fold change between TRPV2‐si versus control (FC): Q1 (< 0.667), Q2 (0.667–0.769), Q3 (1.3–1.5), and Q4 (> 1.5) (Figure S5A). Based on their related peptides, we performed KEGG functional enrichment analysis and found silencing TRPV2 influenced multiple pathways related to MSC metabolism or response to external stimulus (Figure S5B).

We conducted centrality analysis of differentially expressed succinylated proteins with the STRING database and identified hub genes within the PPI network (Figure 4F). The MCC algorithm highlighted the network diagram of the 10 candidates with the highest linkage relations (Figure 4G). Based on the results in Figure S4, proteins with significantly reduced succinylation levels attracted our attention and were displayed with heatmap (Figure 4H). Among them, YWHAZ caught our attention, which was hub genes in Figure 4F,G, and its succinylation modification significantly decreased in the TRPV2‐si group. YWHAZ, a member of the 14‐3‐3 protein family, interacts with various molecules and plays crucial intracellular roles, including the modulation of autophagy (Mack et al. 2012; Medina et al. 2007). Immunoprecipitation (IP) assays confirmed that changes in TRPV2 expression positively affect the succinylation of YWHAZ in MSCs (Figure 4I and Figure S5C). Additionally, YWHAZ's succinylation was upregulated by HS treatment, further implicating the participation of YWHAZ in HS‐induced alterations of MSCs (Figure S5D,E).

### 2.5 Succinylation of YWHAZ K11 Promotes Autophagy and Stimulates EV Release in HS‐Treated MSCs

**FIGURE 5:**
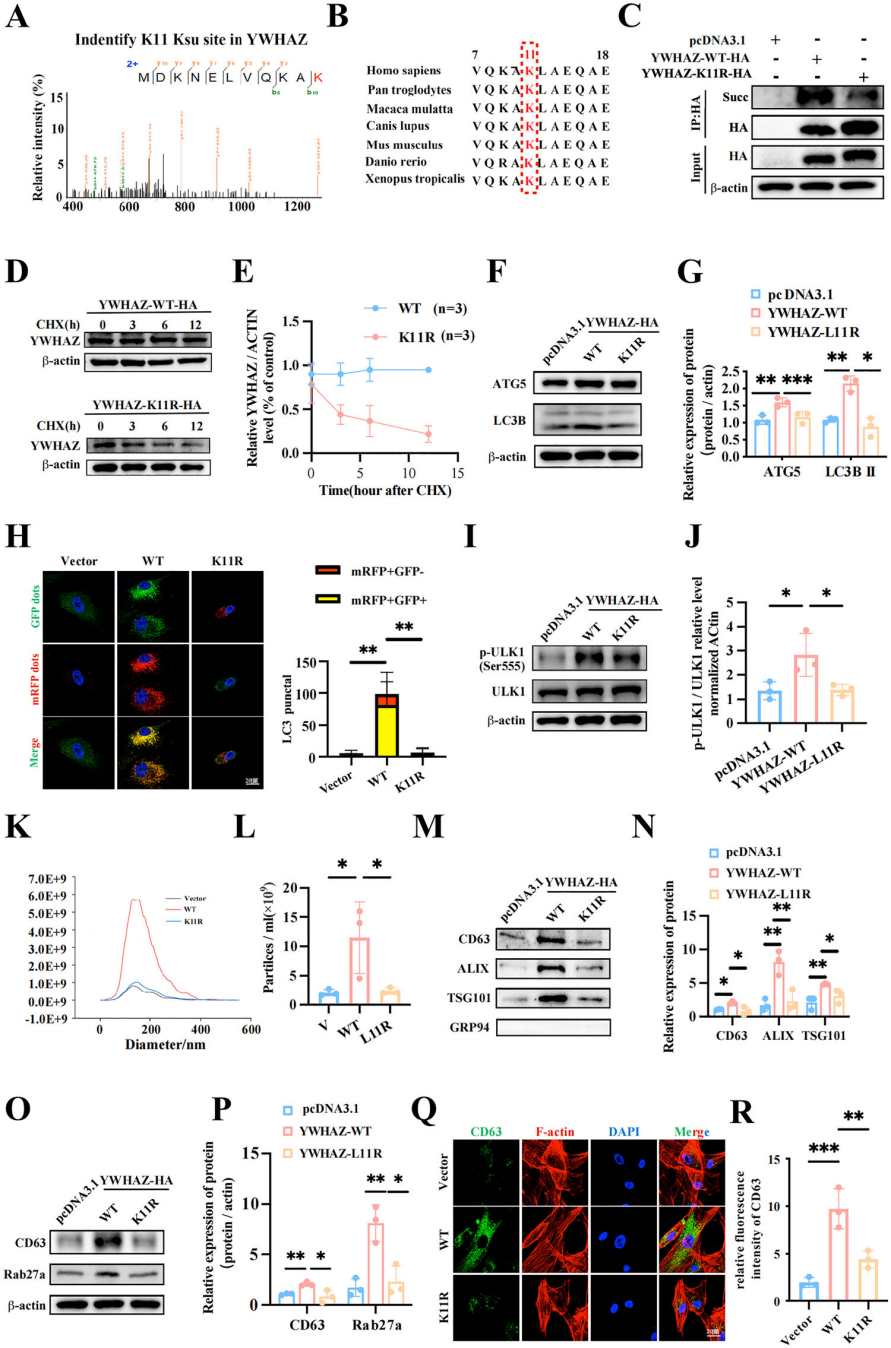
**Succinylation of YWHAZ K11 Promotes Autophagy and Stimulates EVs Release in HS‐Treated MSCs**. (A) The MS/MS spectrum of the modified YWHAZ peptide. (B) The sequences surrounding K11 in YWHAZ among seven species were aligned. Lysine 11 of YWHAZ was coloured in red. (C) Western blotting assays showing the influence of Lysine 11 of YWHAZ on its Succinyl modification in MSCs. (D) A CHX (10 µM) chase assay was used to compare the stability of the wild‐type YWHAZ (YWHAZ‐WT) and YWHAZ‐K11R mutant proteins in MSCs. (E) The line chart showing the degradation of YWHAZ‐WT and YWHAZ‐K114R mutant and the relative protein levels were calculated (*n* = 3). (F, G) Compared with YWHAZ‐WT upregulated the protein levels of ATG5 and LC3B in MSCs and the relative protein levels were calculated. YWHAZ‐K11R induced the opposite changes. (H) Compared with YWHAZ‐WT, YWHAZ‐K11R decreased and increased the autophagosomes, respectively. Scale bar = 20 µm. (I, J) Western blotting assays showing the effects of YWHAZ on the phosphorylation of ULK1 and the relative protein levels were calculated. (K, L) The isolated EVs of each group were examined by NTA and the particle concentration was analysed. (M, N) EV‐related proteins were assessed by western blotting in indicated groups and the relative protein levels were calculated. (O, P) Compared with YWHAZ‐WT, YWHAZ‐K11R decreased the expression of CD63 and Rab27a in MSCs and the relative protein levels were calculated. (Q, R) Compared with YWHAZ‐WT, YWHAZ‐K11R had opposite effects on the CD63 and the relative fluorescence intensity was analysed. All experiments of EV collection were subjected to a standardized 48‐h incubation protocol. Data are shown as the mean ± SD (*n*  =  3). *p < 0.05, **p < 0.01, ***p < 0.001.

Next, we evaluated whether YWHAZ's succinylation could influence autophagy and EV release in HS‐treated MSCs. LC‐MS/MS analysis showed YWHAZ's succinylation mainly occurred at lysine site 11 (K11) (Figure 5A), a conserved site across species (Figure 5B). Previous studies have shown that mutating K to arginine (R) can simulate hyposuccinylation. We constructed the inactive‐mutant YWHAZ (YWHAZ‐K11R) (Figure S6A) and proved the downregulation of YWHAZ's succinylation significantly shortened its half‐life (Figure 5C,E and Figure S6B).

Additionally, western blotting assays showed that YWHAZ significantly increased Atg5 levels and the LC3II/I ratio, which were inhibited by the K11 inactive mutation, suggesting that YWHAZ's succinylation influenced its regulation on autophagy‐related proteins (Figure 5F,G). The mRFP‐GFP‐LC3 reporter assays further validated the effects of YWHAZ's succinylation on the autophagosome formation of MSCs (Figure 5H). Previous studies presented that YWHAZ stimulated autophagy by promoting the phosphorylation of ULK1 (Choudhery et al. 2015) or Beclin11 (Abd‐El‐Aziz et al. 2022), two key proteins involved in initiation of autophagosome formation. Here, we found YWHAZ could bind with ULK1, but not Beclin1 (Figure S6C,D), and increase the phosphorylation of ULK1 at Ser555, and the K11 inactive mutation significantly blocked this effect (Figure 5I,J), which suggested that YWHAZ‐induced autophagosome formation depended on its regulation of the phosphorylation of ULK1. Moreover, we demonstrated that YWHAZ promotes EV release from MSCs, which was reversed by the K11 inactive mutation (Figure 5K–R). Collectively, these results indicate that YWHAZ's succinylation at K11 participates in HS‐induced autophagy and subsequent EV release in MSCs.

### 2.6 HS Treatment Regulates Sacculation of YWHAZ in MSCs via Activating SUCLG2 and OXCT1

**FIGURE 6:**
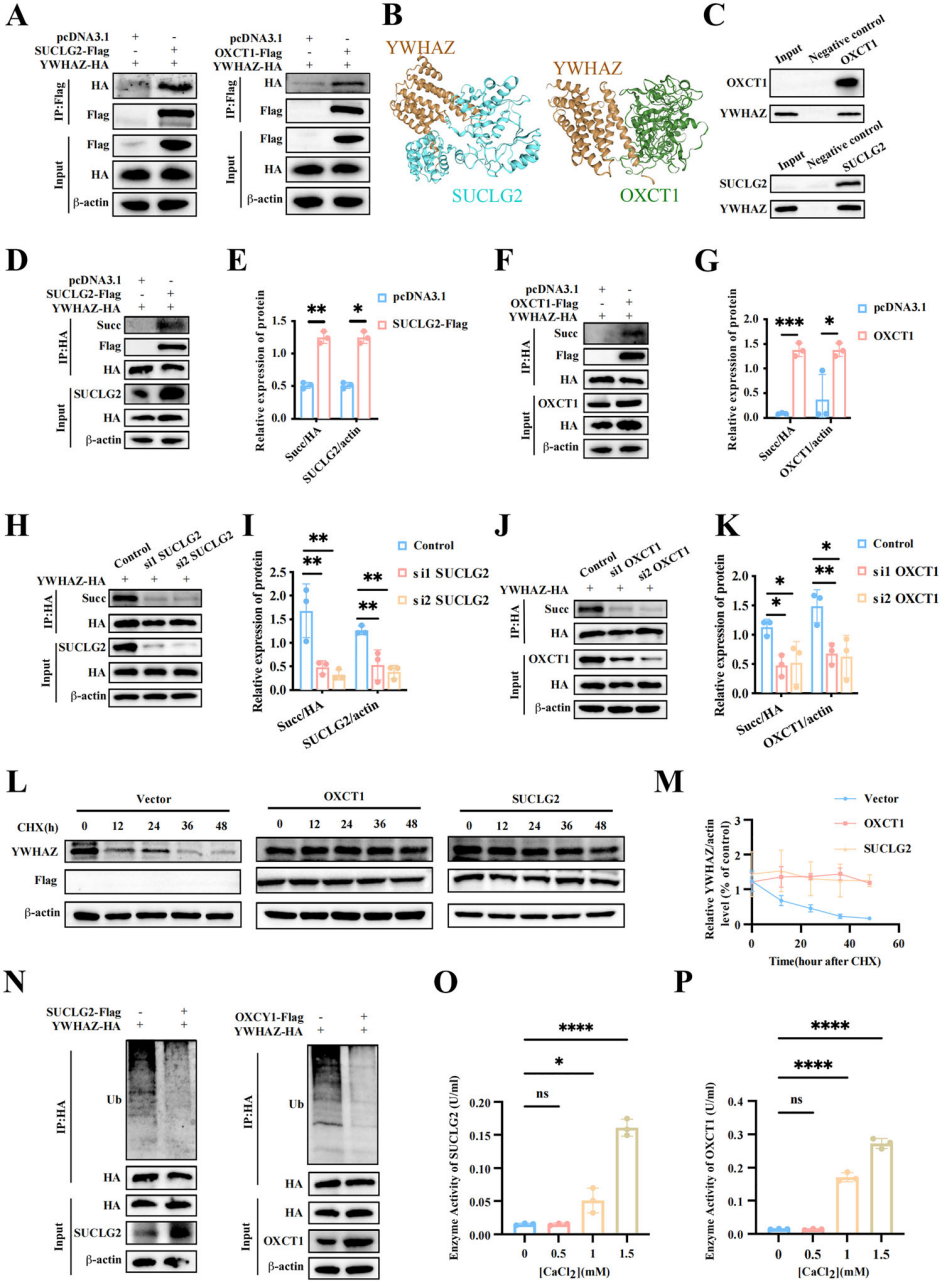
**HS Treatment Regulates Succinylation of YWHAZ in MSCs via Activitiing SUCLG2 and OXCT1**. (A) The interaction between SUCLG2 or OXCT1 and YWHAZ was investigated by immunoprecipitation. (B) Protein docking model of the interaction between SUCLG2 or OXCT1 and YWHAZ. (C) Purified YWHAZ‐GST recombinant protein was immobilized on Glutathione‐Sepharose beads and incubated with SUCLG2 or OXCT1 recombinant protein, followed by immunoblotting. (D–G) SUCLAG2 and OXCT1 increased Succ modification of YWHAZ and the relative Succ modification levels were calculated. (H–K) Knockdown of SUCLG2 or OXCT1 decreased Succ modification of YWHAZ and the relative Succ modification levels were calculated. (L) Quantification of protein stability after CHX treatment in MSCs transfected with SUCLG2 or OXCT1. (M) The relative protein levels of YWHAZ with indicated treatments (*n* = 3). (N) Effect of SUCLG2 and OXCT1 on the ubiquitination of YWHAZ. (O, P) Effect of the concentration of CaCl_2_ on the enzyme activity of SUCLG2 and OXCT1. Data are shown as the mean ± SD (*n*  =  3). *p < 0.05, ****p < 0.001.

Succinylation was regulated by various succinyls, including succinyl‐coenzyme A (CoA) synthetase ADP‐forming subunit beta (SUCLA2), succinate‐CoA ligase GDP‐forming subunit beta (SUCLG2), succinate dehydrogenase (SDHA), lysine acetyltransferase 2A (KAT2A), 3‐oxoacid CoA‐transferase 1 (OXCT1), and sartain 5 (SIRT5) (C. Wang et al. 2024). We conducted IP assays in MSCs to analyse associations between YWHAZ and these enzymes. Only SUCLG2 and OXCT1 could bind to YWHAZ (Figure 6A and Figure S7A–D), which were further predicted using the HDOCK server (http://hdock.phys.hust.edu.cn/) (Figure 6B). And GST pull‐down assays confirmed the direct binding of YWHAZ to SUCLG2 and OXCT1 (Figure 6C).

Further experiments showed that overexpressing or silencing SUCLG2 and OXCT1 significantly regulated YWHAZ's sacculation (Figure 6D–K). Figure 5D,E showed that YWHAZ's succinylation at K11 could prolong its protein's half‐life. We then examined the influence of SUCLG2 and OXCT1 on YWHAZ stability and proved that both enzymes significantly extended YWHAZ's half‐life (Figure 6L,M), which was mainly attributed to YWHAZ's succinylation, which could significantly suppress its ubiquitination (Figure 6N). Was the YWHAZ's succinylation mediated by TRPV2 related to SUCLG2 and OXCT1? Given that TRPV2 is a calcium‐permeable channel, we speculated that TRPV2‐induced Ca^2+^ influx under HS treatment may upregulate the activity of SUCLG2 and OXCT1. As shown in Figure 6O,P and Figure S7E, increasing concentrations of Ca^2+^ indeed elevated the activity of both enzymes. Collectively, these results indicate that HS treatment induces Ca^2+^ influx in MSCs, subsequently activating SUCLG2 and OXCT1 to promote YWHAZ's succinylation.

### 2.7 HS Treatment Strengthens the Hepatoprotective Potential of MSCs via Enhanced EV Release

**FIGURE 7:**
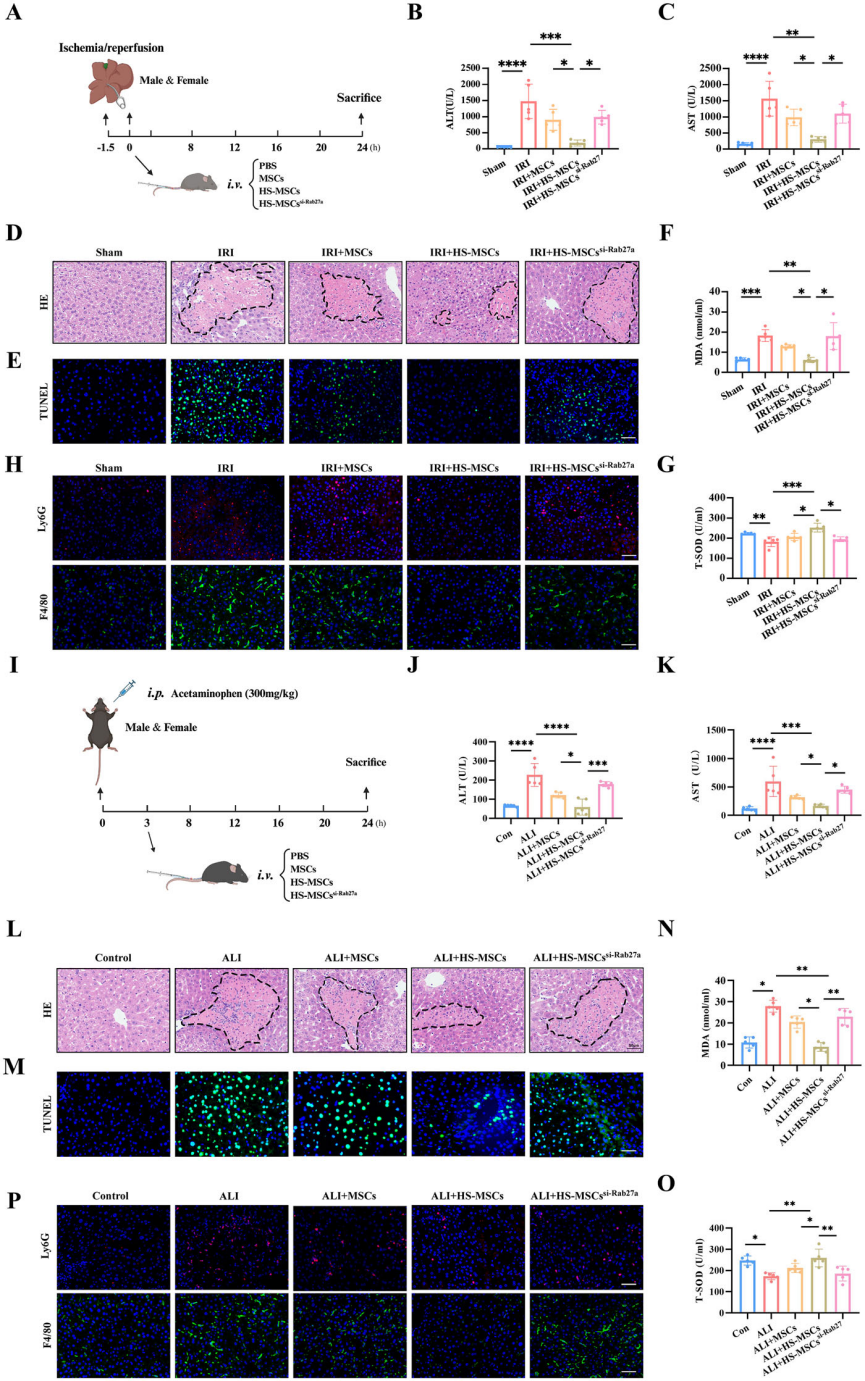
**HS treatment strengthens the hepatoprotective potentials of MSCs in the male liver injury models via enhancing EVs release**. (A) Schematic diagram of hepatic ischemic/perfusion model and MSC treatment strategy. (B) Serum ALT in Sham, IRI, IRI+MSCs, IRI+HS‐MSCs and IRI+HS‐MSCs^siRab27a^. (C) Serum AST in each group. (D) Representative images of H&E staining (the dotted line indicate areas of necrosis) on liver sections from different groups (scale bar: 50 µm); The severity of liver injury was evaluated from histological section and scored according to Suzuki's injury criteria. (E) Representative images of TUNEL staining on liver sections from different groups (scale bar: 50 µm); statistical analyses of the percent of TUNEL‐positive cells in each group. (F) Serum MDA in each group. (G) Serum T‐SOD in each group. (H) Representative images of Ly6G and F4/80 staining on liver sections from different groups (scale bar: 50 µm); Statistical analyses of the percent of Ly6G‐positive or F4/80‐positive cells in each group. (I) Schematic diagram of acetaminophen induced liver injury model and MSC treatment strategy. (J) Serum ALT in Control, ALI, ALI+MSCs, ALI+HS‐MSCs and ALI+HS‐MSCs^siRab27a^. (K) Serum AST in each group. (L) Representative images of H&E staining (the dotted line indicate areas of necrosis) on liver sections from different groups (scale bar: 50 µm); The severity of liver injury was evaluated from histological section and scored according to Suzuki's injury criteria. (M) Representative images of TUNEL staining on liver sections from different groups (scale bar: 50 µm); Statistical analyses of the percent of TUNEL‐positive cells in each group. (N) Serum MDA in each group. (O) Serum T‐SOD in each group. (P) Representative images of Ly6G and F4/80 staining on liver sections from different groups (scale bar: 50 µm); statistical analyses of the percent of Ly6G‐positive or F4/80‐positive cells in each group. Data are shown as the mean ± SD (*n*  =  5). *p < 0.05, **p < 0.01, ***p < 0.001, ****p < 0.001.

To determine whether HS treatment enhances the therapeutic potential of MSCs through increased EV yield, we established two liver injury models, HIRI and APAP‐induced liver injury. First, DiR dye was used to label HS‐treated MSCs and MSCs to investigate their biodistribution in these two models. The results showed that HS‐treated MSCs and MSCs were gathered in the lung and liver, especially in the lung, at the 6th and 24th h after administration, while they were mainly concentrated in the liver after 48 h (Figure S8A–F). Meanwhile, the fluorescent intensity in the liver in the HS‐treated MSCs group was significantly higher than the MSCs group, hinting that HS treatment also strengthens the chemotaxis capacity of MSCs targeting damaged tissue (Figure S8B,E). Previous studies have demonstrated a close correlation between Rab27a expression levels and cellular exosome secretion capacity, where knockdown of Rab27a resulted in a 50%–70% reduction in exosome release (Cabrera et al. 2012; Halade et al. 2010; Peinado et al. 2012; Wang, Li, Bojmar, et al. 2023). Then, we transfected MSCs with si‐Rab27a to limit EV release (Figure S9A). Subsequently, the siRab27a‐transfected MSCs were subjected to HS treatment (1 × 10^6^ cells/mouse) and administered to the two liver injury mouse models in both male and female mice (Figure 7A,I).

As shown in Figure 7B–D, J–L, Figure S9B,E, and Figure S10A–D, and S10K–N, HS‐treated MSCs exhibited stronger hepatoprotective potential than untreated MSCs, as evidenced by the reduced serum hepatic enzymes (ALT and AST), improvements in hepatic pathological changes (including reduced inflammatory cell infiltration, necrosis areas, and hepatic lobule disorders), and lower Suzuki scores. While silencing Rab27a significantly abolished these hepatoprotective effects. Terminal deoxynucleotidyl transferase dUTP nick end labeling (TUNEL) staining, indicating cell apoptosis, showed a similar trend across the liver injury groups (Figure 7E,M; Figure S9C,F, and Figure S10E,F and S10O,P). Malondialdehyde (MDA) and superoxide dismutase (SOD) assays also indicated that HS treatment enhances MSCs’ ability to combat oxidative stress via increased EV release (Figure 7F,G and 7N,O; Figure S10G,H and S10Q,R). Additionally, compared to the MSC group, the numbers of macrophages (F4/80+) and neutrophils (Ly6G+) were significantly decreased in the HS‐treated MSC group, which were reversed with silencing Rab27a (Figure 7H,P; Figure S9D,G and Figure S10I,J and S10S–T).

Collectively, these data reveal that HS treatment enhances the hepatoprotective potential of MSCs by upregulating EV yield.

### 2.8 HS Treatment Heightened the Hepatoprotective Potentials of MSC‐EVs

Next, we also investigated whether HS treatment also heightened the hepatoprotective potentials of MSC‐EVs. The EVs were isolated from HS‐treated MSCs and MSCs labelled with DiR dye, which were respectively administered to two liver injury models mentioned above to detect the in vivo distribution. The results showed that no matter HS treatment or normal control, MSC‐EVs were mainly concentrated in the liver after 24 h of administration, and the fluorescent intensity in the HS‐treated MSC‐EVs group was higher than that in the MSC‐EVs group (Figure S11A,B).

**FIGURE 8:**
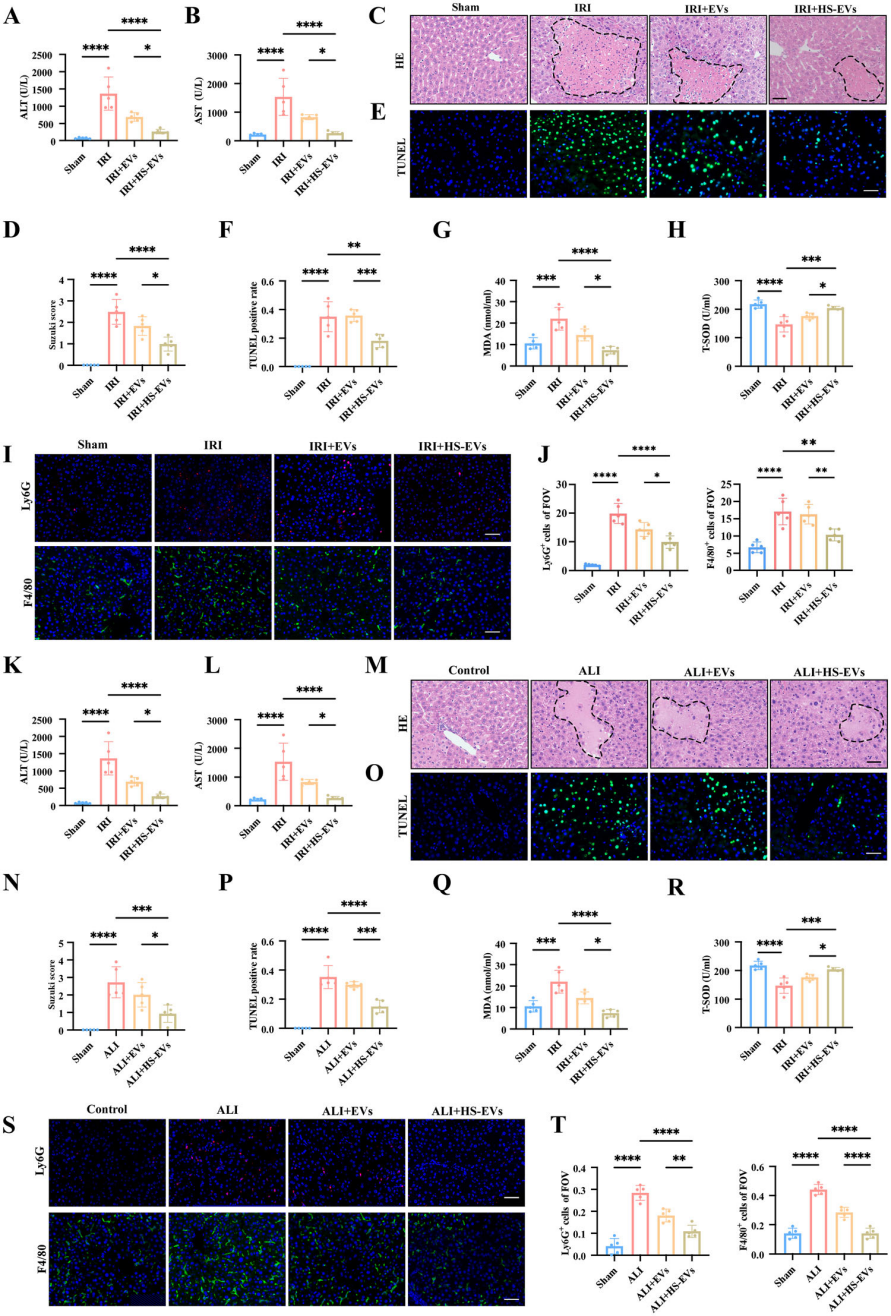
**HS Treatment Heightened the Hepatoprotective potentials of MSC‐EVs in the male liver injury models**. (A, B) Serum ALT and AST in Sham, IRI, IRI+MSCs, IRI+EVs and IRI+HS‐EVs. (C, D) Representative images of H&E staining (the dotted line indicate areas of necrosis) on liver sections from different groups (scale bar: 50 µm); the severity of liver injury was evaluated from histological section and scored according to Suzuki's injury criteria. (E, F) Representative images of TUNEL staining on liver sections from different groups (scale bar: 50 µm); Statistical analyses of the percent of TUNEL‐positive cells in each group. (G, H) Serum MDA and T‐SOD in each group. (I, J) Representative images of Ly6G and F4/80 staining on liver sections from different groups (scale bar: 50 µm); statistical analyses of the percent of Ly6G‐positive or F4/80‐positive cells in each group. (K, L) Serum ALT and AST in Control, ALI, ALI+MSCs, ALI+EVs and ALI+HS‐EVs. (M, N) Representative images of H&E staining (the dotted line indicate areas of necrosis) on liver sections from different groups (scale bar: 50 µm); The severity of liver injury was evaluated from histological section and scored according to Suzuki's injury criteria. (O, P) Representative images of TUNEL staining on liver sections from different groups (Scale bar: 50 µm). (Q, R) Serum MDA and T‐SOD in each group; statistical analyses of the percent of TUNEL‐positive cells in each group. (S, T) Representative images of Ly6G and F4/80 staining on liver sections from different groups (scale bar: 50 µm); statistical analyses of the percent of Ly6G‐positive or F4/80‐positive cells in each group. Data are shown as the mean ± SD (*n*  =  5). *p < 0.05, **p< 0.01, ***p < 0.001, ****p < 0.001.

To detect their therapeutic effects, we used these two kinds of MSC‐EVs to treat the liver injury mouse models in both male and female backgrounds. Compared with the MSC‐EVs group, HS‐treated MSC‐EVs treatment presented more strengthened hepatoprotective potentials, as reflected by the alterations of serum hepatic enzymes, hepatic pathological features, and hepatocellular apoptosis (Figure 8A–F and 8K–P and Figure S12A–F and S12K–P). In addition, the effects of HS‐treated MSC‐EVs on anti‐oxidative stress and immunomodulation were stronger than the MSC‐EVs group, as evidenced by the further increased level of SOD and the reduction of MDA level and macrophages and neutrophils intrahepatic infiltration (Figure 8G–J and 8Q–T and Figure S12G–J and S12Q–T). Taken together, these results presented that HS treatment also heightened the hepatoprotective potential of MSC‐EVs.

### 2.9 Differences in the Composition of Proteins and Non‐Coding RNAs (ncRNAs) in EVs From MSCs and HS‐Treated MSCs

Both proteins and small non‐coding RNAs (sacra), including microRNAs (miRNA), pre‐miRNA, transfer RNA (tRNA), tsRNA, and small nucleolar RNA (snoRNA), have been reported to play important roles in regulating MSC‐EVs’ functions. To depict the differences in proteins and sncRNA expression profiles between two groups, we respectively conducted a Liquid Chromatography Mass Spectrometry (LC‐MS) analysis and a microarray analysis.

**FIGURE 9:**
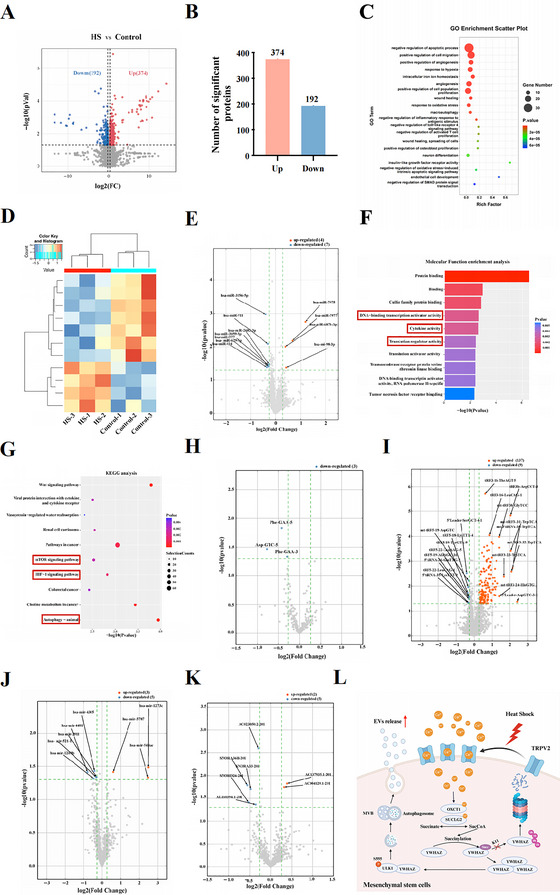
**Distinct protein and non‐coding RNAs (ncRNAs) profiles in EVs from MSCs and HS‐treated MSCs**. (A) Volcano plot was also used to display the different proteins between in the indicated groups. (B) Bar charts showing the differentially expressed proteins in the indicated groups. (C) Bubble chart showing the enriched signalling pathways in ‘HS‐EVs’ vs. ‘Control‐EVs’. (D) The heatmap showed the different miRNA in each group. (E) Volcano plot was also used to display the different miRNAs between two groups. *X* axis shows the log2 fold change and the y axis the log10 p value. Significantly different miRNA are coloured red and blue and labelled. (F) GO enrichment analysis of the genes which were the downstream targets of the 11 significantly expressed miRNAs in HS treated MSCs. (G) KEGG pathway analysis of the genes which were the downstream targets of the four significantly upregulated miRNAs in HS treated MSCs. (H) Volcano plot comparing tRNA fragments between control and HS treated MSCs EVs. Significantly different tRNAs are coloured blue and labelled. (I) Volcano plot comparing tsRNA fragments between control and HS treated MSCs EVs. Significantly different tsRNAs are coloured red and blue and labelled. (J) Volcano plot comparing premiRNA fragments between control and HS treated MSCs EVs. Significantly different premiRNAs are coloured red and blue and labelled. (K) Volcano plot comparing snoRNA fragments between control and HS treated MSCs EVs. Significantly different snoRNAs are coloured red and blue and labelled. (L) Schematic diagram depicting that HS elevates TRPV2 expression, which induces Ca^2+^ influx and regulates YWHAZ succinylation at site K11 via activating SUCLG2 and OXCT1. The succinylation of YWHAZ enhances its stability, which promotes the interaction between YWHAZ and ULK1, enhances ULK1 phosphorylation at site Ser555, and eventually promotes autophagosome formation and EV release.

The results of principal component analysis (PCA) presented that EVs derived from these two groups could be grouped independently, indicating that there were indeed differences between the groups (Figure S13A). As shown in the heatmaps and the expression of multiple proteins in the MSC‐EVs changed with HS treatment (Figure 9A and Figure S13B). A total of 566 proteins were significant differences between these two groups. Among them, 374 were highly expressed in HS‐treated MSC‐EVs, and 192 were low expressed (Figure 9B). Bioinformatic analysis exhibited that compared with the MSC‐EVs group, HS‐treated MSC‐EVs noticeably enriched signalling pathway associating with ‘negative regulation of apoptotic process’, ‘positive regulation of angiogenesis’, ‘response to hypoxia’, ‘intracellular iron ion homeostasis’, ‘positive regulation of cell population proliferation’, ‘macroautophagy’, ‘negative regulation of inflammatory response to antigenic stimulus’, ‘negative regulation of activated T cell proliferation’, ‘negative regulation of toll‐like receptor 4 signalling pathway’, ‘insulin‐like growth factor receptor activity’, and ‘negative regulation of oxidative stress‐induced intrinsic apoptotic signalling pathway’, suggesting that HS treatment may significantly strengthen the roles of MSC‐EVs in promoting tissue regeneration, anti‐oxidative stress, inhibiting cellular apoptosis, and immunoregulating (Figure 9C).

In addition, a total of 11 miRNAs were significantly different between two groups. Seven miRNAs were higher in MSC‐EVs, while four were higher in HS‐treated MSC‐EVs (Figure 9D, 9E). The four most abundant miRNAs in both groups were hsa‐miR‐98‐3p, hsa‐miR‐7977, hsa‐miR‐7975, and hsa‐miR‐6871‐3p, whose levels were higher in HS‐treated MSC‐EVs (Figure S13C). Based on the downstream targets of 11 differentially expressed miRNAs, we performed Gene Ontology (GO) functional analysis. The ‘biological process’ analysis showed these targets were related to transcription and biological development in EVs, such as ‘regulation of cellular metabolic process’, ‘cellular biosynthetic process’ and ‘positive regulation of biological process’ (Figure S13D). In terms of ‘molecular function’, these targets were mainly participants in ‘DNA‐binding transcription activator activity’, ‘cytokine activity’ and ‘translation regulator activity’ (Figure 9F). Additionally, KEGG pathway analysis also revealed that the signalling pathways of ‘autophagy’, ‘mTOR signalling pathway’, and ‘HIF‐1 signalling pathway’ were enriched in the HS‐treated MSC‐EVs (Figure 9G).

Apart from miRNAs, we also analysed the alteration in the composition of tRNAs, tsRNAs, pre‐miRNAs and snoRNAs between two groups. Three tRNAs were more abundant in MSC‐EVs, including Phe‐GAA‐3, Phe‐GAA‐5, and Asp‐GTC‐5 (Figure 9H). Nine tsRNAs were abundant in MSC‐EVs, while 137 were abundant in HS‐treated MSC‐EVs, with the top 10 being 5'Leader‐AspGTC, 3'tiRNA‐mtTrpTCA, three variants of 3'tRF‐mtTrpTCA, et al. (Figure 9I). Eight pre‐miRNAs were differentially abundant between two groups, with three more abundant in the HS‐treated MSC‐EVs (hsa‐mir‐548ac, hsa‐mir‐1273c, and hsa‐mir‐5787) and five more abundant in MSC‐EVs (Figure 9J). Finally, snoRNA profiling showed seven snoRNAs were differentially abundant in the two groups and two snoRNAs were more abundant in HS‐treated MSC‐EVs, namely AC137935.1‐201 and AC004129.1‐201 (Figure 9K). The functions of these different tRNAs, tsRNAs, pre‐miRNAs and snoRNAs between two groups need to be further explored in the future.

## 3 Discussion

Increasing studies have demonstrated MSC‐EVs have the potential to replace MSCs for the treatment of multiple diseases, including HIRI (Lu et al. 2022), retinal IRI (Mathew et al. 2019), acute lung injury (Zhao et al. 2022), and osteoarthritis (B. Yin et al. 2022; You et al. 2023). Additionally, MSC‐EVs can also serve as drug delivery systems for nanotherapy. (Zou et al. 2023) The therapeutic efficacy of engineered EVs has been explored in Alzheimer's disease (Xu et al. 2022; T. Yin et al. 2023), cardiovascular diseases (Lai et al. 2022), and cancer therapy (Zhang, Zhang, Gu, et al. 2021), achieving satisfactory results. However, the insufficient yield of EVs severely restricts their clinical application prospects (Dixson et al. 2023). In this study, we demonstrated for the first time that HS could increase EV release from MSCs by upregulating autophagosome formation. This is partially attributed to the promotion of TRPV2 expression by HS, which triggers calcium accumulation and activates SUCLG2 and OXCT1 to increase YWHAZ's succinylation, succinylation ultimately enhancing the phosphorylation of ULK1.

HS treatment has been identified as an effective approach to enhance the synthesis of self‐protective proteins and stimulate the biological functions of cells. Chen X, et al. reported HS‐treated MSCs could inhibit ovarian granulosa cells’ apoptosis and enhance MSCs’ repair efficacy in chemotherapy‐induced premature ovarian failure (Chen et al. 2018). Q. Wang et al. demonstrated HS improves the survival of MCSs in a chemotherapy environment via elevating HSP70 and HSP90 expression (Wang et al. 2019). T. Yang et al. found exosomes derived from HS‐treated MSCs displayed greater efficacy in alleviating cisplatin‐induced ototoxicity in mice (Yang et al. 2022). Our previous study also showed that HS enhances the pulmonary protective potential of MSCs by upregulating HSP70 expression (Lv et al. 2021). Whether HS has other effects on MSCs remains to be further investigated. Until now, no specific approach has been reported to effectively enhance the EV yield of MSCs. Only Z. Liao et al. found metformin can facilitate EV release from MSCs and optimize therapeutic efficacy in intervertebral disc degeneration (Liao et al. 2021). In this study, we demonstrated that HS could promote EV release. Given that HS is easy to perform and low‐cost, this approach has significant potential for clinical translation. Moreover, we confirmed the therapeutic efficacy of HS‐treated MSCs in two liver injury models and demonstrated that these effects are partially attributed to the enhanced yield of EVs.

In addition to heat‐shock proteins, TRPV2, an evolutionary conserved non‐selective calcium‐permeable ion channel, was also found to be elevated in MSCs under HS treatment (Uhlén et al. 2015). TRPV2 regulates multiple cellular processes, including thermosensation, phagocytosis, mechanosensation, and osmosensation (Li et al. 2024; Li et al. 2023; Link et al. 2010), which play pivotal roles in both healthy and cancer cells, such as maintaining physiological cardiomyopathy, placental development, T‐cell activation, and inducing drug resistance and metastasis (Pumroy et al. 2022; W. Zhang et al. 2022). The function of TRPV2 in MSCs under HS has not been investigated. In this study, we found TRPV2 can increase autophagosome formation by activating calcium flux, thereby promoting HS‐mediated EV release. Overexpressing or silencing TRPV2 in MSCs significantly affected HS‐mediated EV release. Additionally, intervention in autophagosome formation almost reversed TRPV2‐induced EV release.

External stress, such as HS, can trigger transient changes in the modification of intracellular proteins (Cheng et al. 2023; Sheng et al. 2024). To reveal the mechanism of HS‐mediated EV release via TRPV2, we examined the effects of HS and TRPV2 on the modification of intracellular proteins. Five common protein modifications were detected: phosphorylation, acetylation, succinylation, lactylation, and SUMOylation. Among these, the alteration in succinylation was the most significant. And HS significantly increased intracellular protein succinylation, while silencing TRPV2 downregulated it. YWHAZ is one of the most significantly modified molecules in this process, and its stability is increased with succinylation. YWHAZ, a member of the 14‐3‐3 protein family (Mhawech 2005), can serve as an adaptor to regulate multiple cellular signalling pathways (Kim et al. 2021). In this study, YWHAZ can bind to ULK1, a key protein in autophagy initiation, and enhance its phosphorylation at site Ser555, thereby promoting autophagosome formation and increasing EV release.

YWHAZ has primarily been studied for its binding partners and the functions of these interactions, with little attention paid to the regulation of YWHAZ stability. In this work, we identified for the first time that succinylation of YWHAZ at K11, mediated by SUCLG2 and OXCT1, can enhance YWHAZ stability by inhibiting ubiquitin‐proteasome‐mediated degradation. So how does TRPV2 regulate the succinylase activity of SUCLG2 and OXCT1? Triggering calcium influx is a classical function of TRPV2. However, it remains unclear whether calcium ions can affect the succinylase activity of SUCLG2 and OXCT1. We performed in vitro enzyme activity assays and demonstrated calcium ions can significantly increase the succinylase activity of SUCLG2 and OXCT1.

In conclusion, our work presents HS treatment can upregulate EV yield in MSCs. Mechanistically, HS elevates TRPV2 expression, which induces Ca^2+^ influx and regulates YWHAZ succinylation at site K11 via activating SUCLG2 and OXCT1. The succinylation of YWHAZ enhances its stability, which promotes the interaction between YWHAZ and ULK1, enhances ULK1 phosphorylation at site Ser555, and eventually promotes autophagosome formation and EV release (Figure 9L). These findings provide new insights for promoting the clinical translational application of MSC‐EVs in the future.

## 4 Methods and Materials

### 4.1 Generation and Culture of Human MSCs

The preparation of MSCs was ethically approved by the Research Ethics Committee of the Third Affiliated Hospital of Sun Yat‐sen University. MSCs were harvested and cultured in this investigation in accordance with accepted practices (Perico et al. 2017). All procedures were conducted in a sterile environment. To outline the process briefly, consenting donors provided fresh UCs, whcih were cut into 10 mm^3^ pieces and digested. The cells were given DMEM low‐glucose medium (Gibco, Life, Australia).

### 4.2 HS Treatment

In vitro HS experiments, cells were seeded in either 6‐well plates or T175 tissue culture flasks, with the medium refreshed upon reaching a cell density of 70%–80%. The cells were then exposed to HS conditions in a 42°C water bath for 1 h, followed by incubation at 37°C, 5% CO_2_, and a humidified atmosphere for 12 or 48 h.

### 4.3 EVs Isolation and Quantification

Consistent with prior EV isolation protocols (Liu et al. 2024; Wang, Wu, Zhai, et al. 2023), cells were cultured to 70%–80% confluency before medium replacement. Post‐replacement, the experimental group underwent immediate HS (1 h), whereas controls received no intervention. EVs from both groups were collected after 48 h of continued culture. The conditioned media was gathered and centrifuged at 300 × *g* for 10 min to eliminate floating cells. Any leftover cells or debris were then removed by centrifuging it at 3000 × *g* for 30 min. Subsequently, the supernatant underwent centrifugation at 100,000 × *g* for 70 min at 4°C to isolate EVs. Next, the supernatant was discarded, and the MSC‐EVs were washed with PBS at 100,000 × *g* at 4°C for 70 min. Finally, the pellet was resuspended in PBS to obtain MSC‐EVs. The EVs can either be stored at −80°C or proceed to further experiment.

### 4.4 NTA

To measure the particle sizes and concentrations, the MSC‐derived EVs were evaluated through NTA employing a NanoSight NS300 system (Germany, ZetaView) and subsequently analysed using NTA 3.2 software.

### 4.5 TEM

The EV solution was pipetted onto copper grids and left at 25°C for 3 min. Following the removal of excess suspension using filter paper, the EVs were stained with 5–10 µL of phosphotungstic acid at 25°C for 3 min. Subsequently, images were taken using an electron microscope (FEI Spirit T12 120 kV, USA).

### 4.6 Western Blotting

EVs and cellular proteins were collected, and western blotting analysis was carried out as previously described (Li et al. 2024). The antibodies are list in Table S1.

### 4.7 RNA Extraction and qRT‐PCR

TRIzol (Sigma, USA) was used to extract total RNA in accordance with the manufacturer's instructions. Next, using the Transcriptor First Strand cDNA Synthesis Kit (Promega, USA), RNA was reverse transcribed to cDNA. Subsequent qRT‐PCR was conducted as per the provided guidelines, employing SYBR Green Pro Taq HS Mix. GAPDH served as the internal control. Specific primer details can be found in Table S1.

### 4.8 Immunofluorescence and mRFP‐GFP‐LC3 Immunofluorescence Assay

The cells were fixed with 4% paraformaldehyde (PFA) at room temperature for 20 min, permeabilized using 0.5% Triton X‐100 for 5 min, blocked with 10% BSA in PBS for 30 min, and then subjected to the CD63 antibody staining. Subsequently, secondary antibodies labelled with FITC (Biyuntian, Wuhan, Hubei, China) were employed. DAPI was used for nuclei staining, while the cytoskeleton was treated with Actin‐Tracker Red‐594 (Biyuntian, Wuhan, Hubei, China). The transfection of mRFP‐GFP‐LC3 was carried out following the manufacturer's instructions. After fixation and permeateation, the cells were stained with DAPI and underwent fluorescence microscopy (Zeiss, Germany). And we analysed 5 fields per sample to ensure representative and statistically meaningful results.

### 4.9 Intracellular Ca^2+^ Concentration Assessment

The cells were incubated with Fluo‐4 Staining Solution (Beyotine S106, China) in the dark at 37°C for 30 min. The visualization of intracellular Ca^2+^ concentrations was carried out using fluorescence microscopy (Zeiss, Germany).

### 4.10 Plasmids, Transfection, Infection

The TRPV2, YWHAZ‐WT, YWHAZ‐K11R, YWHAZ‐K11E, SUCLG2, SUCLA2, OXCT1, SDHA, KAT2A, SIRT5, and UB overexpression plasmids were purchased from MIAOLING BIOLOGU (Wuhan, China): pEnCMV‐EGFP‐Linker‐TRPV2(human)‐SV40‐Neo(P25682), pLV3‐CMV‐YWHAZ(human)‐3×HA‐Puro(P57396), G37544/pLV3‐CMV‐YWHAZ(human)‐K11R‐3×HA‐Puro, G37544/pLV3‐CMV‐YWHAZ(human)‐K11E‐3×HA‐Puro, pLV3‐CMV‐SUCLG2(human)‐2‐3×FLAG‐CopGFP‐Puro(P50931), pCMV‐SUCLA2(human)‐3×FLAG‐Neo(P45443), pCMV‐OXCT1(human)‐FLAG‐SV40‐Neo(P4044), pCMV‐EGFP‐SDHA(human)‐3×FLAG‐Neo(P60045), pCDNA3.1‐KAT2A(human)‐FLAG(P20785), pCMV‐SIRT5(human)‐3×FLAG‐Neo(P44526), pCDNA3.1‐6×His‐Ubiquitin‐K63(P63706). The siRNA against TRPV2, OXCT1, and SUCLG2 were purchased from GenePharma (Shanghai, China). Transfection and infection procedures were conducted utilizing jetPRIME (polyplus‐transfection) as per the manufacturer's guidelines. Details of all plasmids and siRNA sequences can be found in Table S1.

### 4.11 LC–MS/MS Analysis

LC–MS/MS analysis was carried out as previously described (Gomez‐Zepeda et al. 2024). Briefly, samples were separated using Easy nLC, an HPLC system with nanolitre flow rates. A timsTOF Pro mass spectrometer (Bruker) was used for mass spectrometry analysis following the sample's chromatographic separation.

### 4.12 Pan‐Antibody‐Based PTM Enrichment

Pan‐antibody‐based PTM enrichment was carried out as previously published (Luo et al. 2022). In brief, to enrich modified peptides, the peptides were incubated with anti‐succinyl‐lysine antibody beads, washed, treated with TFA, and desalted using C18 STAGE Tips.

### 4.13 IP Assay

The cell lysates were incubated at 4°C for an overnight period using Anti‐HA and Anti‐FLAG Nanobody Magarose Beads (ApalifeBio, China). Following rinses with 0.05% Tween 20 in PBS and lysis buffer, the beads were mixed with 1× SDS/PAGE loading buffer for subsequent western blotting analysis.

### 4.14 GST‐Pull Down Assay

YWHAZ‐GST protein was incubated with Glutathione‐Sepharose beads (ThermoFisher) for 2 h at 4°C. Following this, the complexes were resuspended in lysis buffer, and GST‐tagged proteins were introduced into the solution. This mixture underwent an overnight incubation at 4°C. Subsequent to washing, the pulled‐down proteins were subjected to analysis via SDS‐PAGE and identified by a western blotting assay.

### 4.15 Measurement of Succinyl Transferase Activity of OXCT1 and SUCLG2

Human recombinant OXCT1 proteins (0.5 µM) or recombinant SUCLG2 proteins (0.5 µM) was incubated with recombinant LDHA protein (0.5 µM) in succinyl transferase assay buffer in the presence of different concentrations of calcium. The production of NADPH was quantitatively analysed with a modified protocol from the Colorimetric Histone Acetyltransferase Activity Assay Kit (ScienCell, Germany). In brief, the recombinant proteins were mixed with 10 µL developer solution (1X), 1 µL NADH generating enzyme, 10 µL WST‐1 solution, 0.2 mM succinyl‐CoA and the buffer is filled to 100 µL. Succinylation of a peptide substrate regulated by succinyl transferase releases the free form of CoA, which can then be applied to create NADH by acting as a coenzyme. Spectrophotometric analysis was used to measure the stoichiometric NADH generation after interacting with WST‐1 at 440 nm. An identical reaction was set up without OXCT1 or SUCLG2 protein and used as the blank control.

### 4.16 Animals

The animals used in this investigation were C57BL/6 background mice (male/female, aged 8–10 weeks), procured from GemPharmatech in Nanjing, China. All animal procedures were conducted in compliance with Chinese regulations concerning experimental animal usage and had received approval from the Institutional Animal Care and Use Committee (IACUC) at Jennio Biotech Co., Ltd. Each segment of the experiment was grouped based on the principle of randomization.

### 4.17 Mouse Liver Injury Models Induced by APAP or HIRI

APAP‐induced liver injury: Mice were administered a single intraperitoneal injection of APAP (300 mg/kg, Sigma Aldrich) in saline to induce liver injury. HIRI: Following anaesthesia with 1% sodium pentobarbital (100 µL/10 g), a non‐invasive arterial clamp was applied to halt blood flow to the left and middle liver lobes for 90 min, inducing ischaemia. Subsequently, the reperfusion phase was then started by removing the clamp. The sham group only had laparotomies. All mice were euthanized 24 h post surgery, and both serum and liver samples were collected for additional examination.

### 4.18 In Vivo Tracing

To supervise the distribution of MSCs, HS‐treated MSCs, MSC‐EVs, and HS‐treated MSC‐EVs in two liver injury models, the samples were stained with the lipophilic dye 1,1’‐dictadecyl‐3,3,3’,3’‐tetramethylindotricarbocyanine iodide (DiR; Thermo Fisher Scientific, USA) following the manufacturer's instructions. In vivo biodistribution was investigated by a Bruker Small Animal Optical Imaging System (In‐Vivo Xtreme II; Billerica, MA). After that, the heart, lung, liver, spleen, and kidneys were collected from each mouse, and the fluorescence intensity in the organs was detected using the same imaging system.

### 4.19 Measurement of Serum Hepatic Enzymes

Serum alanine and aspartate aminotransferase levels were measured with anChemray 800 (Shanghai, China).

### 4.20 Hematoxylin and Eosin Staining (H&E)

Following the preparation of paraffin‐embedded liver sample sections stained with eosin and hematoxylin, the histological features of the liver sample were graded using Suzuki's criteria.

### 4.21 Terminal‐Deoxynucleotidyl Transferase Mediated Nick End Labelling (TUNEL) Assay

The TUNEL assay of frozen sections was carried out as previously published (Li et al. 2024), and the positive regions were observed under a fluorescence microscope (Zeiss, Germany).

### 4.22 Immunofluorescence Staining

Immunofluorescence staining of the liver sample was performed as previously described (Li et al. 2024) and were visualized under a fluorescence microscope (Zeiss, Germany).

### 4.23 Measurement of MDA and Total Superoxide Dismutase (T‐SOD)

The plasma MDA was measured by an MDA assay kit (Nanjing, China). In brief, an aliquot of plasma (or a known volume of plasma protein) was mixed with an equal volume of TBA reagent in a test tube, heat in a boiling water bath or an oven at 95°C for 40 min, and then centrifuged to remove the precipitated proteins. After that, the supernatant was transferred to a cuvette, followed by measuring the absorbance of the pink‐coloured complex at 532 nm optical density (OD) value by a spectrophotometer.

The plasma T‐SOD was measured by a T‐SOD assay kit (Nanjing, China). The plasma sample was mixed with the reaction buffer in test tubes and incubated the reaction mixture at 37°C for 40 min. The reaction was terminated by adding a stopping buffer, and the absorbance of OD value at 450 nm was measured using a spectrophotometer.

### 4.24 Library Preparation and RNA‐seq

Library preparation and RNA‐seq were carried out as previously described (J. Zhang et al. 2023). Briefly, total RNA was isolated, and the quantification and quality of the samples were evaluated using a spectrophotometry and electrophoresis. Each sample's 1–2 µg total RNA was used for RNA sequencing library construction involving oligo dT enrichment, KAPA Stranded RNA‐Seq Library Prep Kit, double‐stranded cDNA synthesis via the dUTP method, library quality assessment with Agilent 2100 Bioanalyzer, qPCR quantification, and sequencing on Illumina HiSeq 4000 sequencer with various steps including NaOH denaturation, in situ amplification, and 150 double‐end cycle sequencing

### 4.25 Bioinformatic and Statistical Analysis

Bioinformatic and statistical analyses were carried out as previously described (Huang et al. 2022). In brief, raw sequencing data from Illumina HiSeq 4000 underwent quality control, trimming, alignment to the reference genome, statistical analysis of alignment results, followed by various analyses including gene and transcript expression quantification, differential gene expression screening, GO and pathway enrichment analyses, and visualization through scatter plots, volcano plots, and cluster plots to aid data interpretation.

### 4.26 Statistics Analysis

All data were statistically analysed and graphically presented using GraphPad Prism 9.0 (GraphPad Software, San Diego, CA, USA). The data are expressed as the means ± standard deviation (SD) and were compared using One‐way analysis of variance (ANOVA). Statistical significance was considered when *p* values were less than 0.05.

## Consent

All authors of this study agreed to publish.

## Conflicts of Interest

The authors declare no conflicts of interest.

## Supporting information



Supporting Information

## Data Availability

All data are available from the corresponding authors upon reasonable request.
