# Supplementary material for: Heat Shock Strengthens the Protective Potential of MSCs in Liver Injury by Promoting EV Release Through Upregulated Autophagosome Formation
Source: J Extracell Vesicles. 2025 May 6;14(5):e70084. doi: 10.1002/jev2.70084 (PMC12053880; doi:10.1002/jev2.70084)
Supplement: Supplementary file 1 — Supporting Information [file JEV2-14-e70084-s001.pdf]

Supporting information

Heat shock strengthens the protective potential of MSCs by promoting EVs release in liver injury via upregulating TRPV2-mediated YWHAZ succinylation

Tingting Wang, Yihang Gong, Huizhu Lin, Xuejiao Li, Jinliang Liang, Xiaofeng Yuan, Cuiping Li, Zhongying Hu, Haitian Chen, Jiaqi Xiao, Jiebin Zhang, Yasong Liu, Chenhao Jiang, Jia Yao, Qi Zhang, Rong Li, Jun Zheng

Table of Contents

Supplementary figures…………………………………………………....2

Supplementary tables…………………………………………………24

Supplementary Figures

Fig. S1

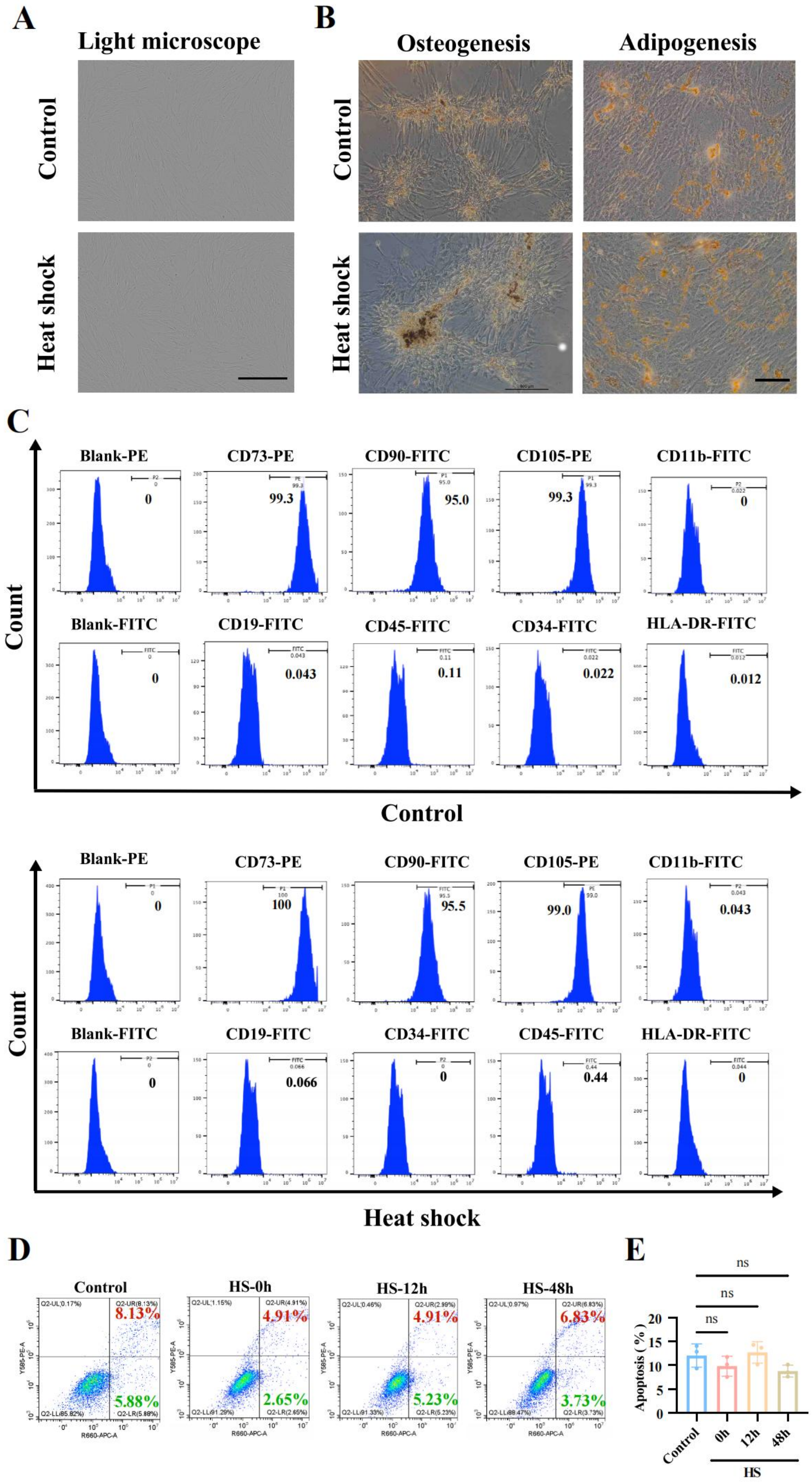

Figure S1. Identification of UC-MSCs and the effects of HS treatment on the properties and viability of MSC.

(A)The morphology of MSCs was detected by light microscopy (scale bar: 100 μm). (B) Representative images of MSCs of each group, which differentiate into osteogenesis and adipogenesis (scale bar: 100 μm). (C) The cell surface makers were determined by flow cytometric analysis, which highly expressed CD73, CD90 and CD105, and negatively expressed CD11b, CD19, CD45, CD34 and HLA-DR. (D-E) The viability of MSC treated with or without HS were assessed by flow cytometric analysis. Data are shown as the mean ± SD (n = 3). *p < 0.05, **p< 0.01, ***p< 0.001, ****p< 0.001.

Fig. S2

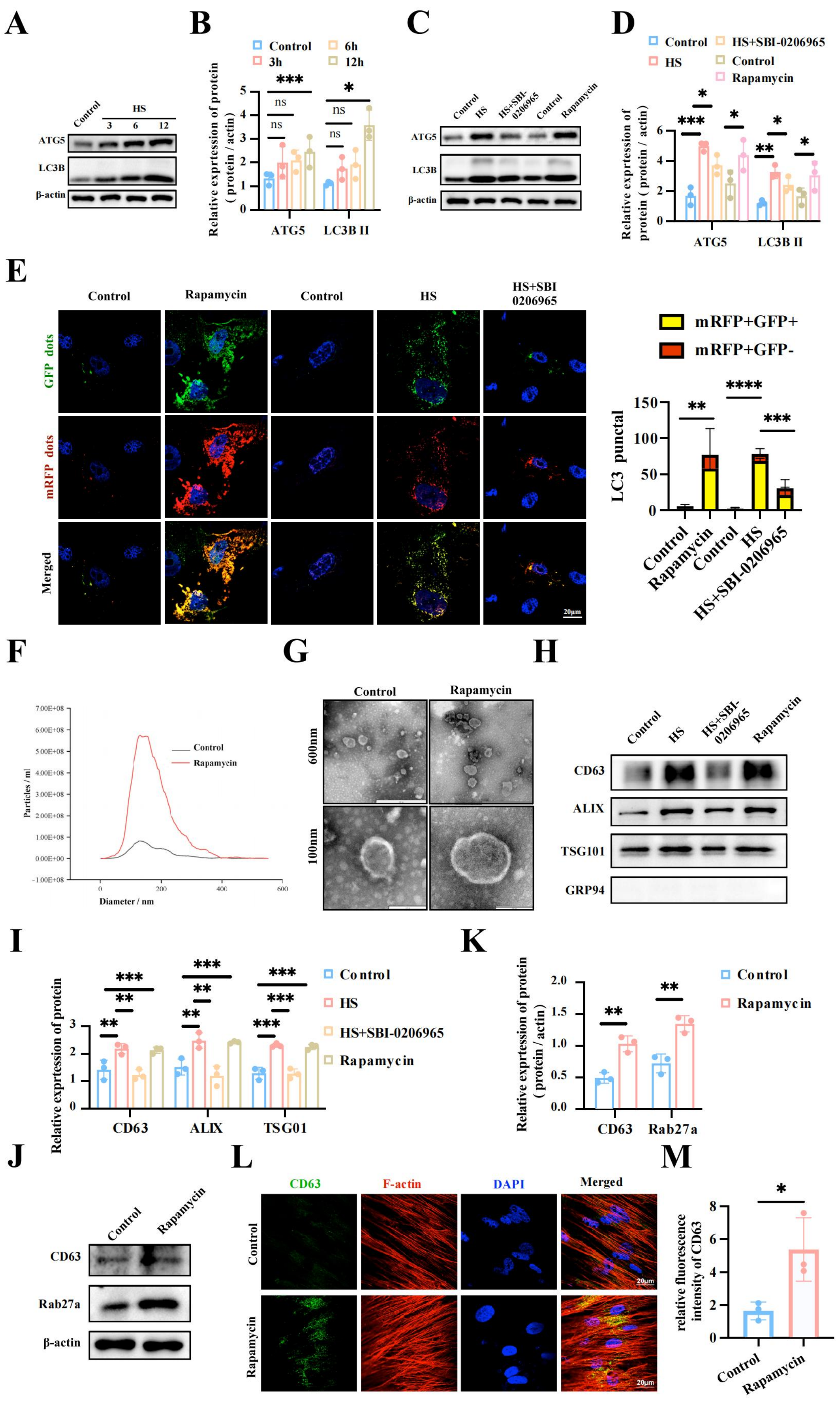
A
Control
HS
3
6
12
ATG5
LC3B
β-actin
B
Control
3h
6h
12h
Relative exprtession of protein (protein / actin)
ATG5
LC3B II
C
Control
HS
HS+SBI-0206965
Control
Rapamycin
ATG5
LC3B
β-actin
D
Control
HS
HS+SBI-0206965
Control
Rapamycin
Relative exprtession of protein (protein / actin)
ATG5
LC3B II
E
Control
Rapamycin
Control
HS
HS+SBI 0206965
GFP dots
mRFP dots
Merged
20μm
mRFP+GFP+
mRFP+GFP-
LC3 punctal
Control
Rapamycin
Control
HS
HS+SBI-0206965
F
Control
Rapamycin
Particles / ml
Diameter / nm
G
Control
Rapamycin
600nm
100nm
H
Control
HS
HS+SBI-0206965
Rapamycin
CD63
ALIX
TSG101
GRP94
I
Control
HS
HS+SBI-0206965
Rapamycin
Relative exprtession of protein
CD63
ALIX
TSG01
K
Control
Rapamycin
Relative exprtession of protein (protein / actin)
CD63
Rab27a
J
Control
Rapamycin
CD63
Rab27a
β-actin
L
CD63
F-actin
DAPI
Merged
Control
Rapamycin
20μm
M
relative fluorescence intensity of CD63
Control
Rapamycin

Figure S2. HS treatment promotes autophagosome formation to enhance EV release from MSCs.

(A-B) Western blotting analysis of autophagy-related proteins in MSCs at different times after HS treatment as indicated and the relative protein levels were calculated. (C-D) Western blotting analysis of autophagy-related proteins in MSCs with indicated treatments and the relative protein levels were calculated. (E) Rapamycin increased the autophagosomes, while SBI-0206965 suppressed the autophagosome formation induced by HS treatment. (Scale bar, 20 μm). (F) The number and size of EVs isolated from indicated MSCs groups were examined by NTA. (G) TEM showed the morphology of indicated EVs (scale bar: 600nm and 100 nm). (H-I) The key proteins of EVs isolated from indicated MSCs groups were assessed by Western blotting and the relative protein levels were calculated. (J-K) Cell lysates from MSCs treated with or without rapamycin were assessed by western blotting and the relative protein levels were calculated. (L-M) Rapamycin had positive effects on the CD63 and the relative fluorescence intensity was analyzed. (scale bar: 20 μm). All experiments of EVs collection were subjected to a standardized 48-hour incubation protocol. Data are shown as the mean ± SD (n = 3). *p < 0.05, **p< 0.01, ***p< 0.001, ****p< 0.001.

Fig. S3

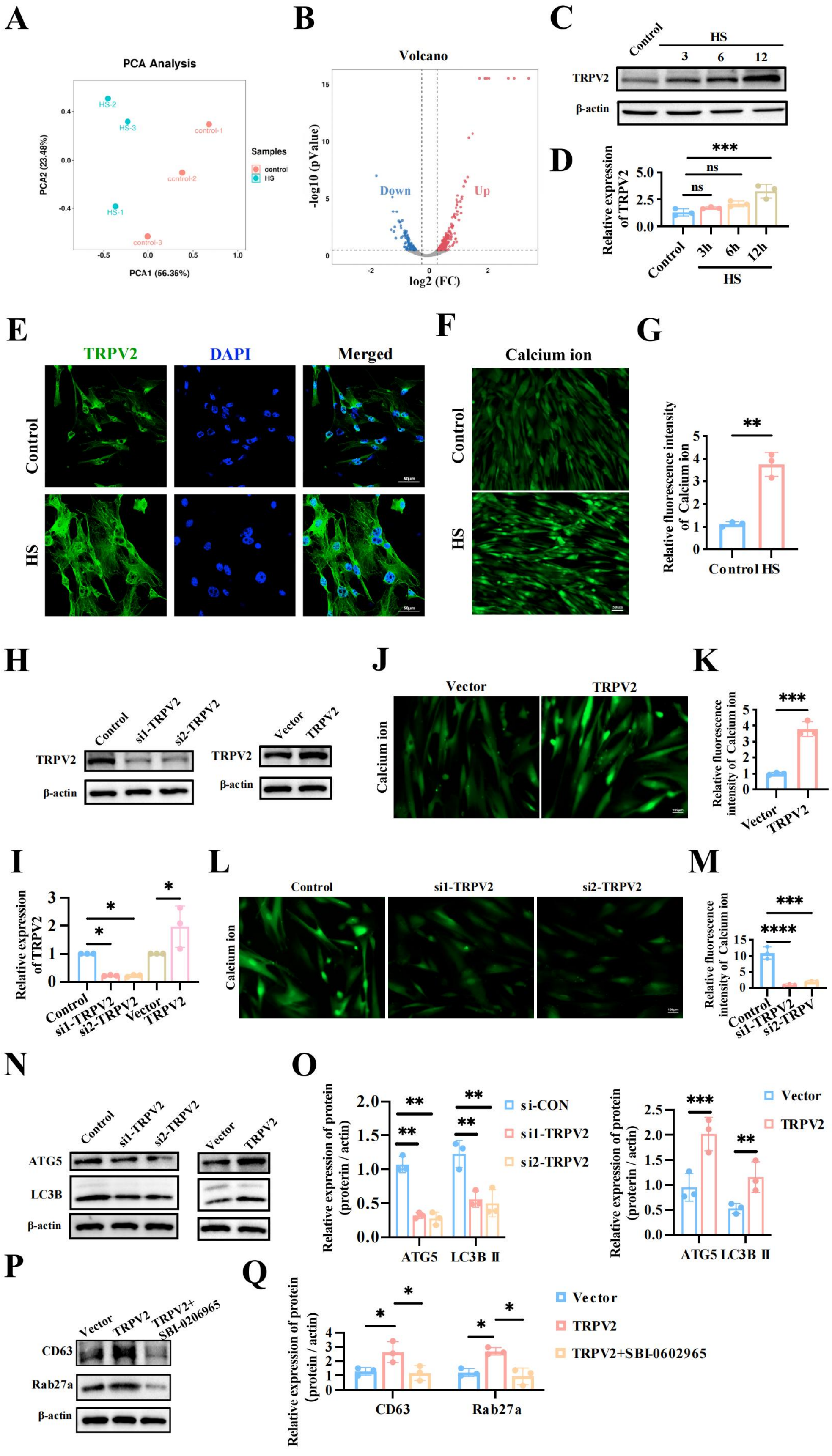
A
PCA Analysis
PCA1 (56.36%)
PCA2 (23.48%)
Samples
control
HS
B
Volcano
-log10 (pValue)
log2 (FC)
Down
Up
C
Control
HS
3
6
12
TRPV2
β-actin
D
Relative expression of TRPV2
ns
ns
***
Control
3h
6h
12h
E
TRPV2
DAPI
Merged
Control
HS
F
Calcium ion
G
Relative fluorescence intensity of Calcium ion
**
Control HS
H
Control
si1-TRPV2
si2-TRPV2
Vector
TRPV2
J
Vector
TRPV2
Calcium ion
K
Relative fluorescence intensity of Calcium ion
***
I
L
Control
si1-TRPV2
si2-TRPV2
M
****
***
N
ATG5
LC3B
β-actin
O
Relative expression of protein (proterin / actin)
si-CON
si1-TRPV2
si2-TRPV2
ATG5
LC3B Ⅱ
Vector
TRPV2
P
TRPV2+ SBI-0206965
CD63
Rab27a
Q
Relative expression of protein (protein / actin)
TRPV2+SBI-0602965

Figure S3. HS treatment activates autophagy to stimulate EV release of MSCs by upregulating TRPV2 expression

(A)The PCA analysis of MSCs treated with or without HS. (B) Volcano plot showed the differentially expressed genes in MSCs treated with or without HS treatment. (C-D) Western blotting showed the expression of TRPV2 in different groups as indicated and the relative protein levels were calculated. (E) Immunofluorescence staining images of DAPI (blue) and TRPV2 (green) between control and HS treated Group (Scale bar, 50 μm). (F-G) Ca^{2+} fluorescence images of MSCs treated with or without HS was analyzed (Scale bar: 100 μm). The qualification of Ca2+ fluorescence intensity was measured. (H-I) Knockdown and overexpression efficiency of TRPV2 were assessed by western blotting and the relative protein levels were calculated. (J-K) Knockdown of TRPV2 decrease intracellular calcium ions (Scale bar: 100 μm). The qualification of Ca^{2+} fluorescence intensity was measured. (L-M) Overexpression of TRPV2 increase intracellular calcium ions (Scale bar: 100 μm). The qualification of Ca^{2+} fluorescence intensity was measured. (N-O) Western blotting showed the expression of autophagy-related proteins in different groups as indicated and the relative protein levels were calculated. (P-Q) Western blotting showed the expression of CD63 and Rab27a in different groups as indicated and the relative protein levels were calculated. All experiments of EVs collection were subjected to a standardized 48-hour incubation protocol. Data are shown as the mean ± SD (n = 3). **p< 0.01, ***p< 0.001, ****p< 0.001.

Fig. S4

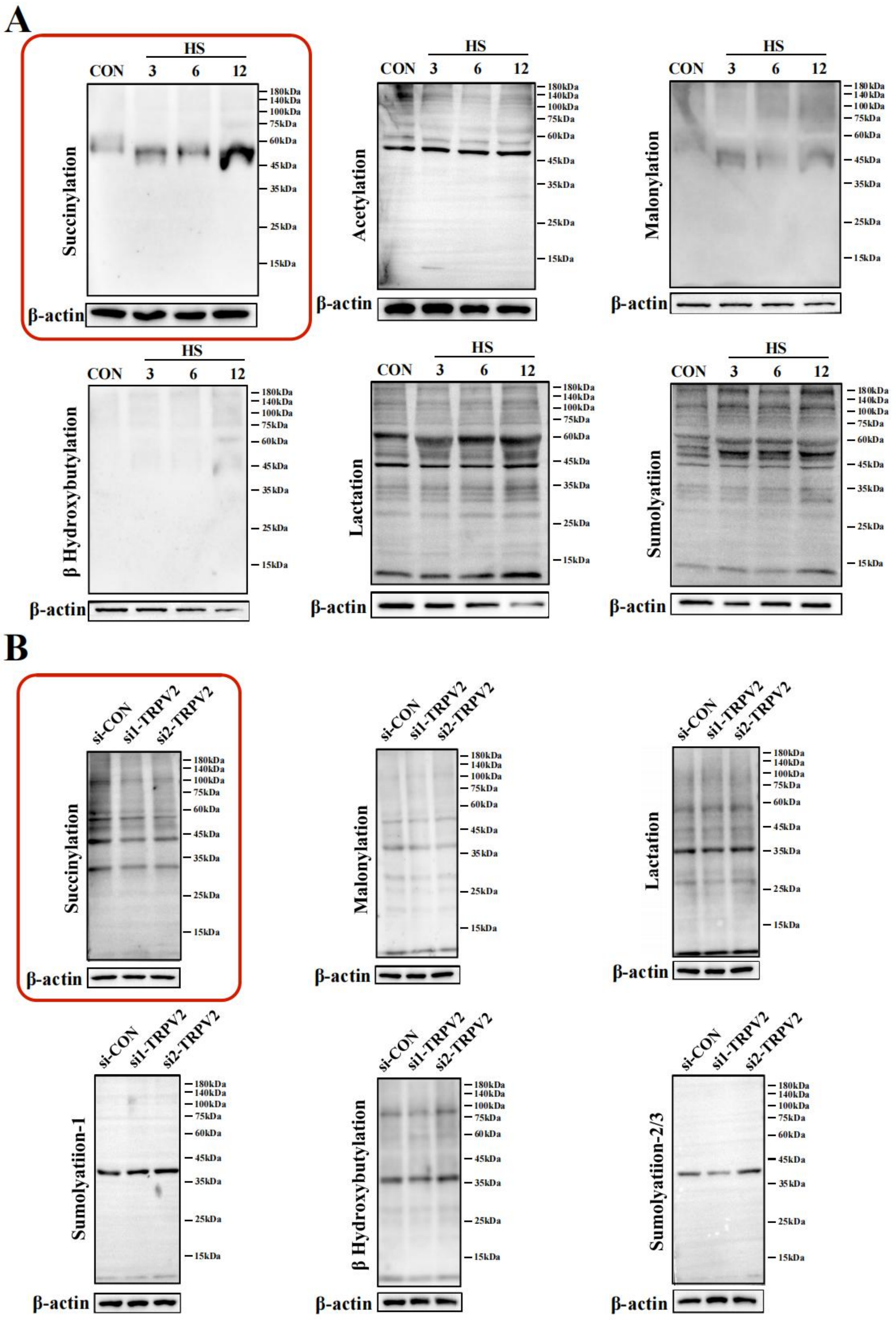

Figure S4. Detection of alterations of common protein modifications in MSCs with indicated treatments.

(A) Comparison of the profiles of the 6 common protein modifications in MSCs treated with or without HS and the level of only succinalytion was significantly increased. (B) Comparison of the profiles of the 6 common protein modifications in MSCs^{siTRPV2} and the level of only succinalytion was significantly decreased.

Fig. S5

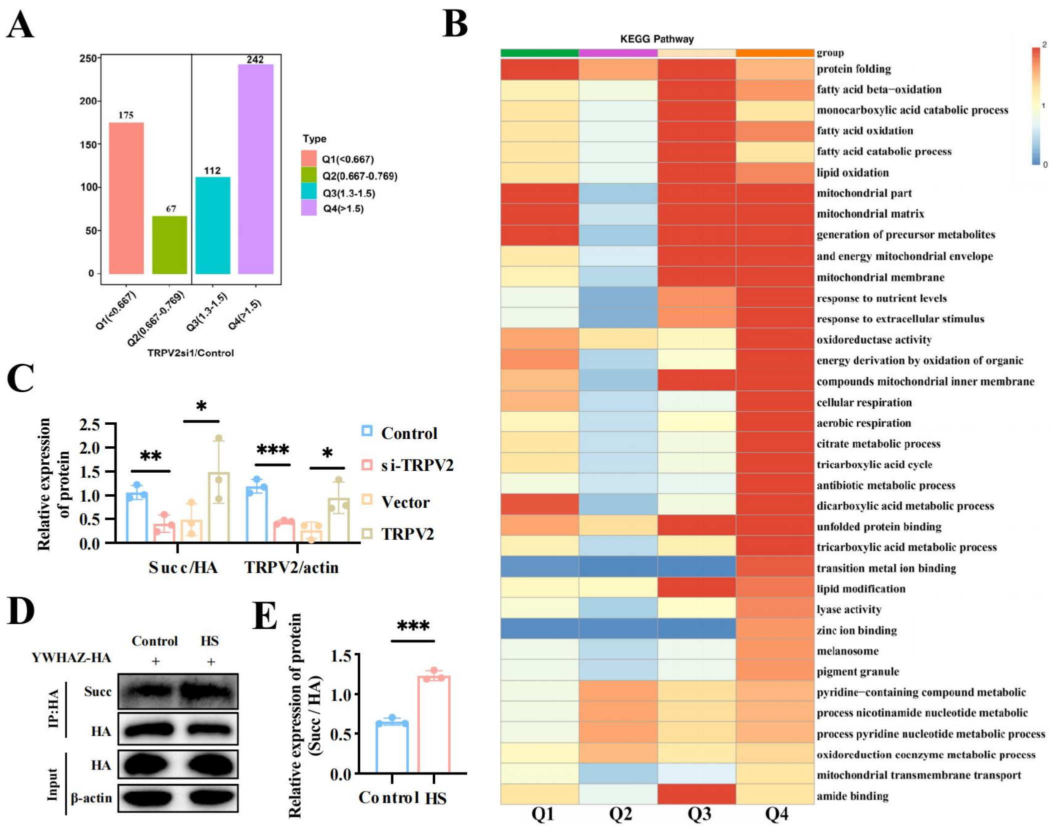

Figure S5. Q classification method, GO enrichment analysis and detection of Succ modification of YWHAZ in MSCs with HS treatment.

(A) The DESPs were classified into four clusters by the Q classification method for further enrichment analysis. (B) GO enrichment analysis showing the enriched functions with DESPs in each Q cluster. (C)The relative protein levels of “Figure. 4I” were calculated. (D-E) HS treatment increased Succ modification of YWHAZ in

MSCs and the relative Succ modification levels were calculated. Data are shown as the mean ± SD (n = 3). **p< 0.01, ***p< 0.001, ****p< 0.001.

Fig. S6

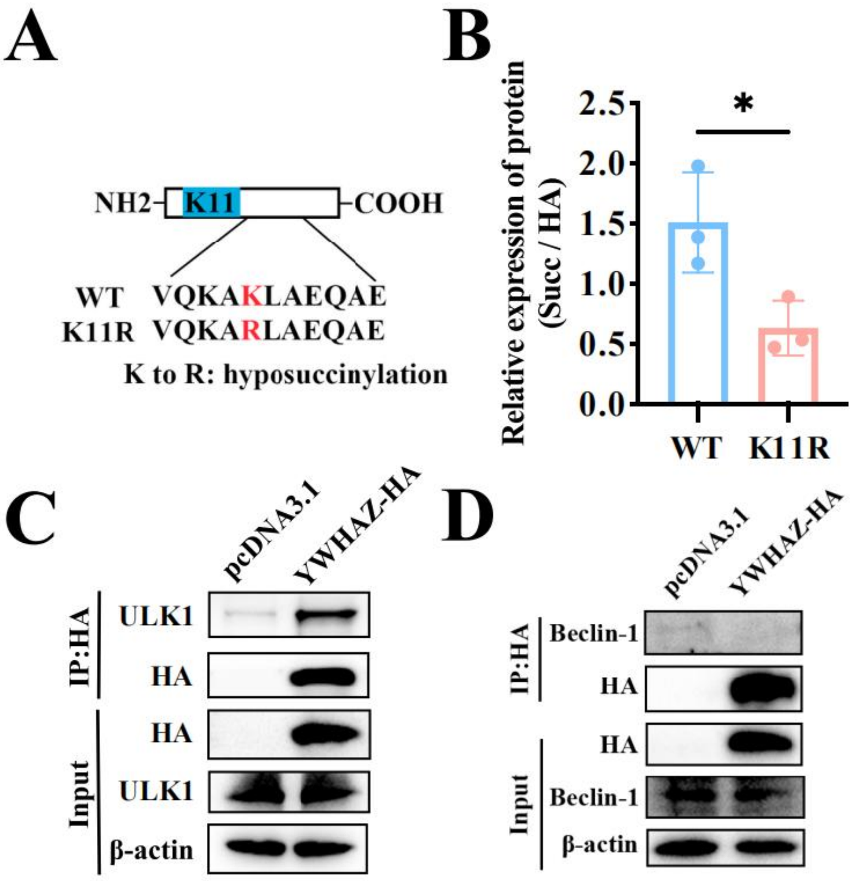

Figure S6. Schematic diagram of mutation and YWHAZ combined with ULK1 instead of Beclin-1.

(A)Schematic diagram of the construction of YWHAZ mutants. (B) The relative protein levels of “Figure. 5C” were calculated. (C) The interaction between YWHAZ and ULK1 was investigated by immunoprecipitation. (D) The interaction between YWHAZ and Beclin-1 was investigated by immunoprecipitation.

Fig. S7

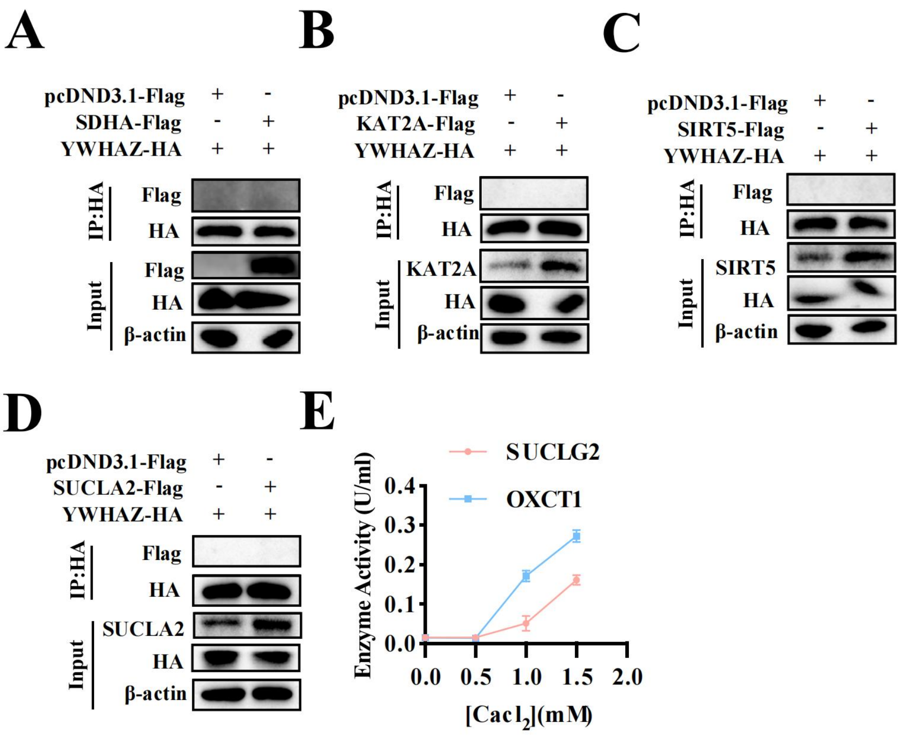

Figure S7. Analysis of the interaction between YWHAZ and enzymes related to succinyl modification and detection of enzyme activity.

(A-D) Indicated succinylases plasmid and YWHAZ-HA expression plasmid were transfected into MSCs, and the cells were then subjected to immunoprecipitation with anti-HA beads to evaluate the interaction between Indicated succinylases and YWHAZ. (E) The concentration of $CaCl_2$ (1mM and 1.5mM) significant affects enzyme activity of OXCT1 and SUCLG2.

Fig. S8

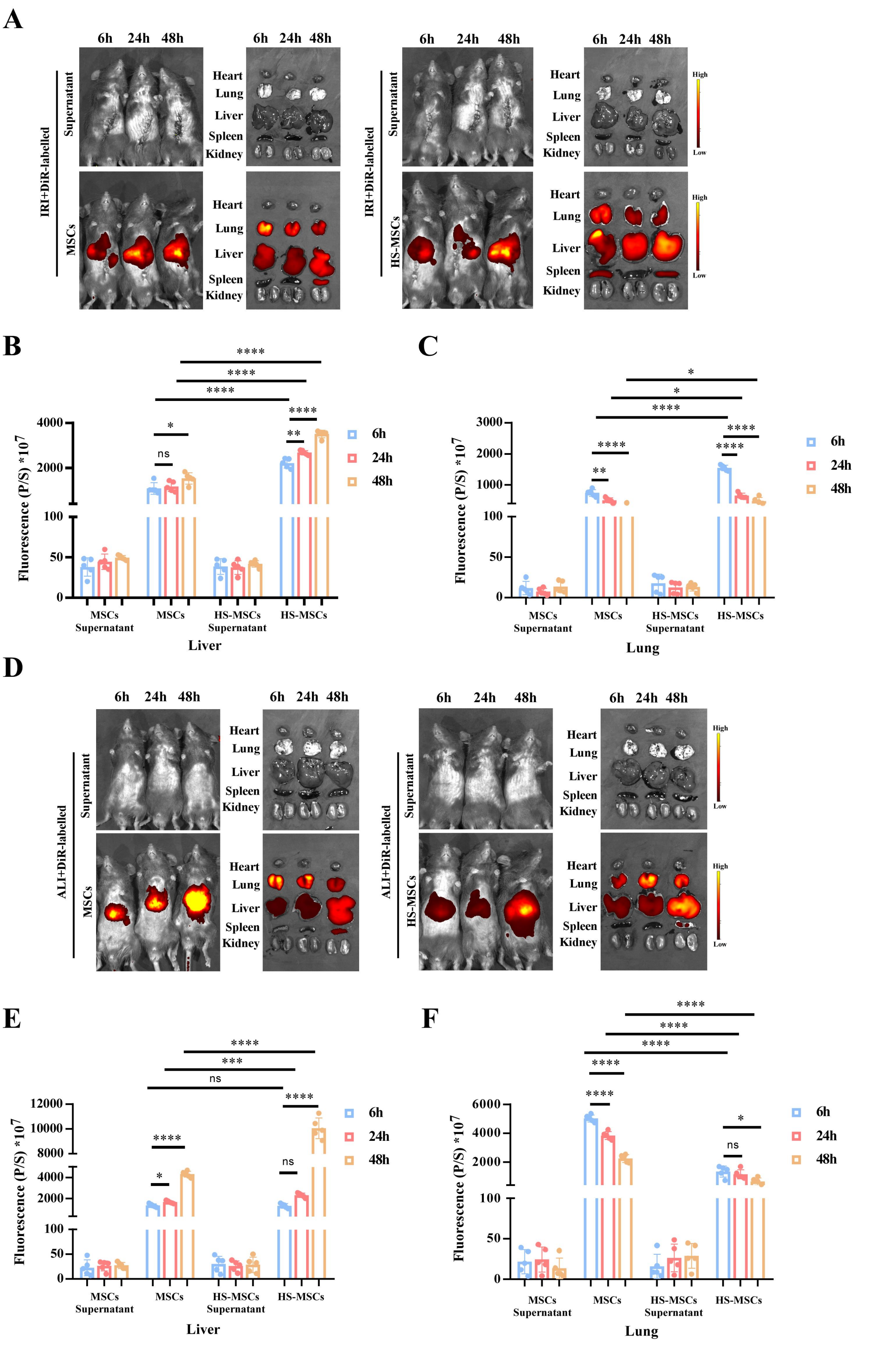

Figure S8. The biodistribution of MSCs and HS-treated MSCs in HIRI and

APAP-induced liver injury mouse models.

(A) Representative IVIS images of 6, 24, and 48 h post-injection of HIRI mouse model injected i.v. with DiR-labeled MSCs supernatant (left, upper), DiR-labeled MSCs (left down), DiR-labeled HS-treated MSCs supernatant (right, upper), or DiR-labeled HS-treated MSCs (right, down) in vivo; Representative IVIS images of five different organs harvested at 6, 24, and 48 h in each group, respectively. (B-C) Quantification of the fluorescent intensity in liver and lung at 6, 24, and 48 h in each group. (D) Representative IVIS images of 6, 24, and 48 h post-injection of APAP-induced liver injury mouse model injected i.v. with DiR-labeled MSCs supernatant (left, upper), DiR-labeled MSCs (left down), DiR-labeled HS-treated MSCs supernatant (right, upper), or DiR-labeled HS-treated MSCs (right, down) in vivo; Representative IVIS images of five different organs harvested at 6, 24, and 48 h in each group, respectively. (E-F) Quantification of the fluorescent intensity in liver and lung at 6, 24, and 48 h in each group. Data are shown as the mean ± SD (n = 5). *p < 0.05, **p< 0.01, ***p< 0.001, ****p< 0.001.

Fig. S9

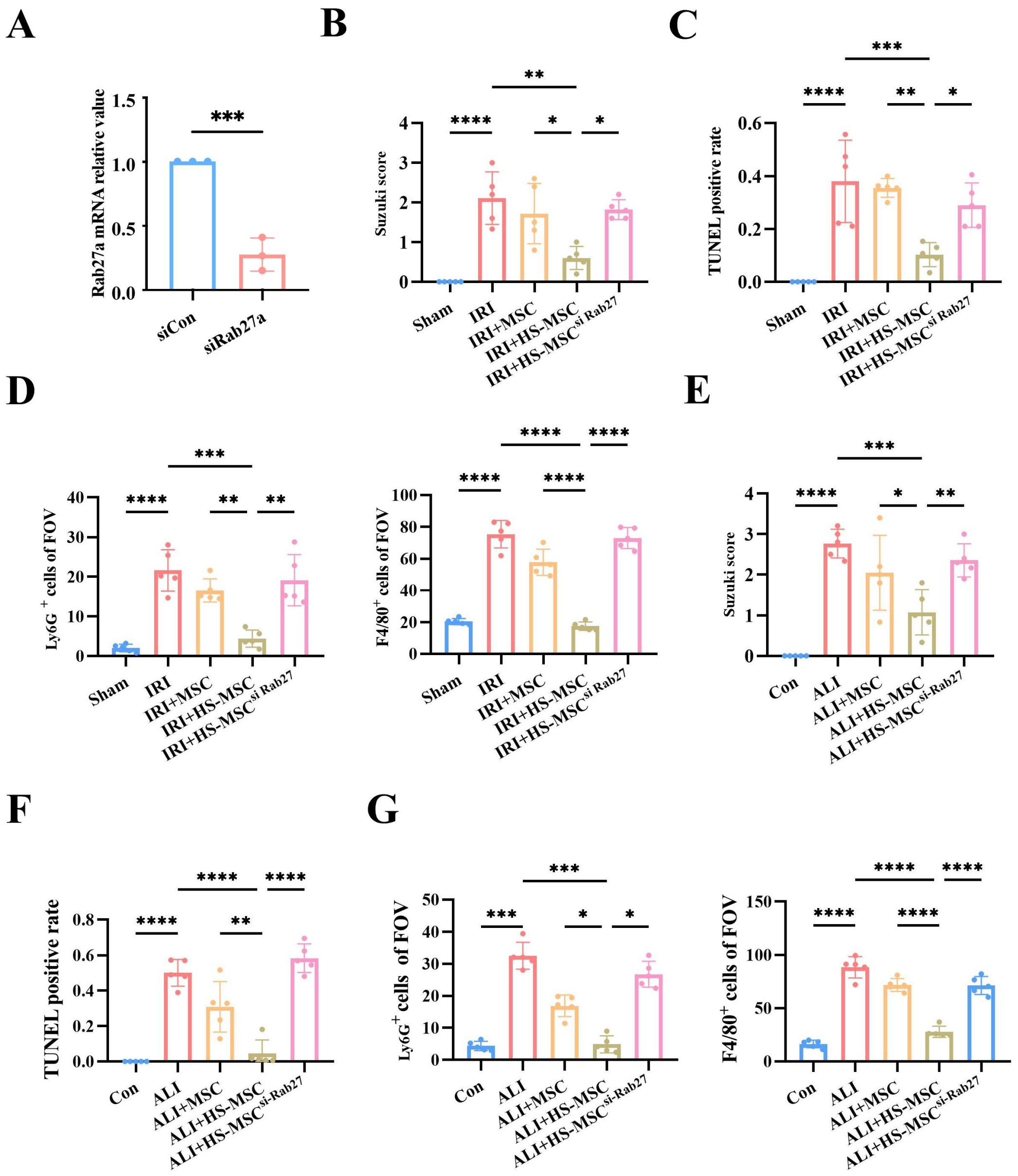

Fig. S9 HS treatment strengthens the hepatoprotective potentials of MSCs in the male liver injury models via enhancing EVs release.

(A) The transfected efficiency of siRab27a were assessed by qPCR. (B) Quantification of liver injury area in Figure 7D. (C) Quantification of TUNEL positive area in Figure 7E. (D) Quantification of Ly6G or F4/80 positive cells infiltration in Figure 7H. (E) Quantification of liver injury area in Figure 7L. (F)

Quantification of TUNEL positive area in Figure M. (G) Quantification of Ly6G or F4/80 positive cells infiltration in Figure 7P. All experiments of EVs collection were subjected to a standardized 48-hour incubation protocol. Data are shown as the mean ± SD (n = 3-5). *p < 0.05, **p< 0.01, ***p< 0.001, ****p< 0.001.

Fig. S10

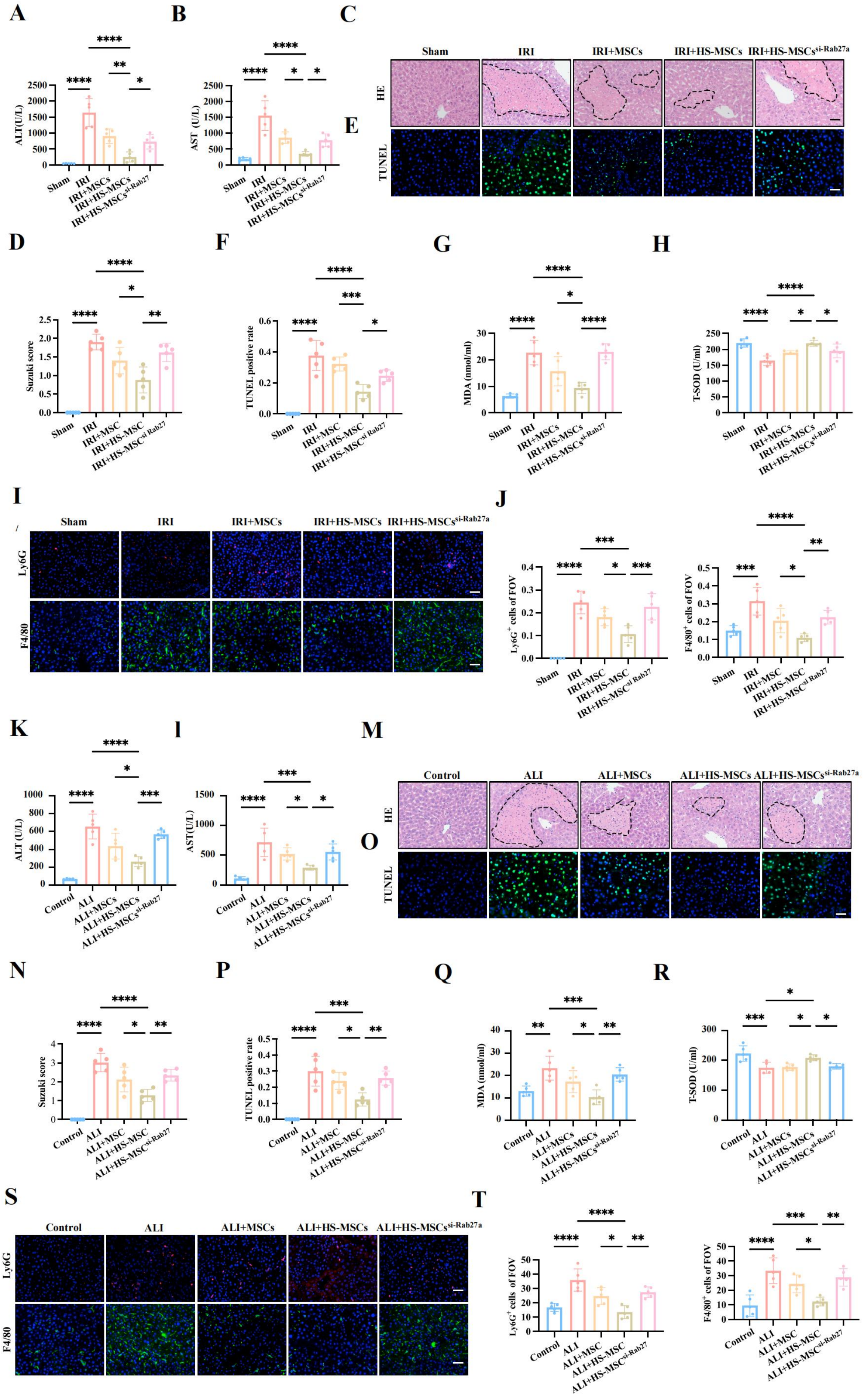

Figure S10. HS treatment strengthens the hepatoprotective potentials of MSCs in

the female liver injury models via enhancing EVs release.

HIRI model: (A) Serum ALT in Sham, IRI, IRI+MSCs, IRI+HS-MSCs and IRI+HS-MSCs^{siRab27a}. (B) Serum AST in each group. (C-D) Representative images of H&E staining (the dotted line indicate areas of necrosis) on liver sections from different groups (Scale bar: 50 μm); The severity of liver injury was evaluated from histological section and scored according to Suzuki's injury criteria. (E-F) Representative images of TUNEL staining on liver sections from different groups (Scale bar: 50 μm); Statistical analyses of the percent of TUNEL-positive cells in each group. (G) Serum MDA in each group. (H) Serum T-SOD in each group. (I-J) Representative images of Ly6G and F4/80 staining on liver sections from different groups (Scale bar: 50 μm); Statistical analyses of the percent of Ly6G-positive or F4/80-positive cells in each group. APAP-induced liver injury model: (K) Serum ALT in Sham, IRI, IRI+MSCs, IRI+HS-MSCs and IRI+HS-MSCs^{siRab27a}. (L) Serum AST in each group. (M-N) Representative images of H&E staining (the dotted line indicate areas of necrosis) on liver sections from different groups (Scale bar: 50 μm); The severity of liver injury was evaluated from histological section and scored according to Suzike's injury criteria. (O-P) Representative images of TUNEL staining on liver sections from different groups (Scale bar: 50 μm); Statistical analyses of the percent of TUNEL-positive cells in each group. (Q) Serum MDA in each group. (R) Serum T-SOD in each group. (S-T) Representative images of Ly6G and F4/80 staining on liver sections from different groups (Scale bar: 50 μm); Statistical analyses of the percent of Ly6G-positive or F4/80-positive cells in each group. Data are shown as the

mean ± SD (n = 5). *p < 0.05, **p< 0.01, ***p< 0.001, ****p< 0.001.

Fig. S11

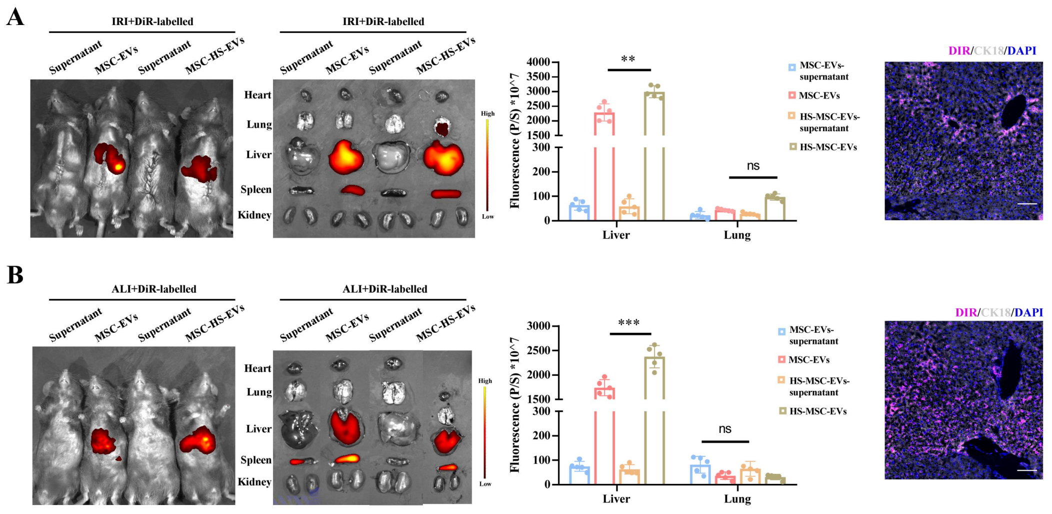

Figure S11. The biodistribution of MSC-EVs and HS-treated MSC-EVs in HIRI and APAP-induced liver injury mouse models.

(A) Representative IVIS images of 24 h post-injection of HIRI mouse model injected i.v. with DiR-labeled MSC-EVs supernatant, DiR-labeled MSC-EVs, DiR-labeled HS-treated MSC-EVs supernatant, or DiR-labeled HS-treated MSC-EVs in vivo (left); Representative IVIS images of five different organs harvested in each group, respectively (second on the left); Quantification of the fluorescent intensity in liver and lung (third on the left); The fluorescent images of the liver following administration of DiR-labeled HS-treated MSC-EVs (right). (B) Representative IVIS images of 24 h post-injection of APAP-induced liver injury mouse model injected i.v. with DiR-labeled MSC-EVs supernatant, DiR-labeled MSC-EVs, DiR-labeled HS-treated MSC-EVs supernatant, or DiR-labeled HS-treated MSC-EVs in vivo (left); Representative IVIS images of five different organs harvested in each group,

respectively (second on the left); Quantification of the fluorescent intensity in liver and lung (third on the left); The fluorescent images of the liver following administration of DiR-labeled HS-treated MSC-EVs (right). Scale bar: 50 μm. Data are shown as the mean ± SD (n = 5). *$p < 0.05$, **$p < 0.01$, ***$p < 0.001$, ****$p < 0.001$.

Fig. S12

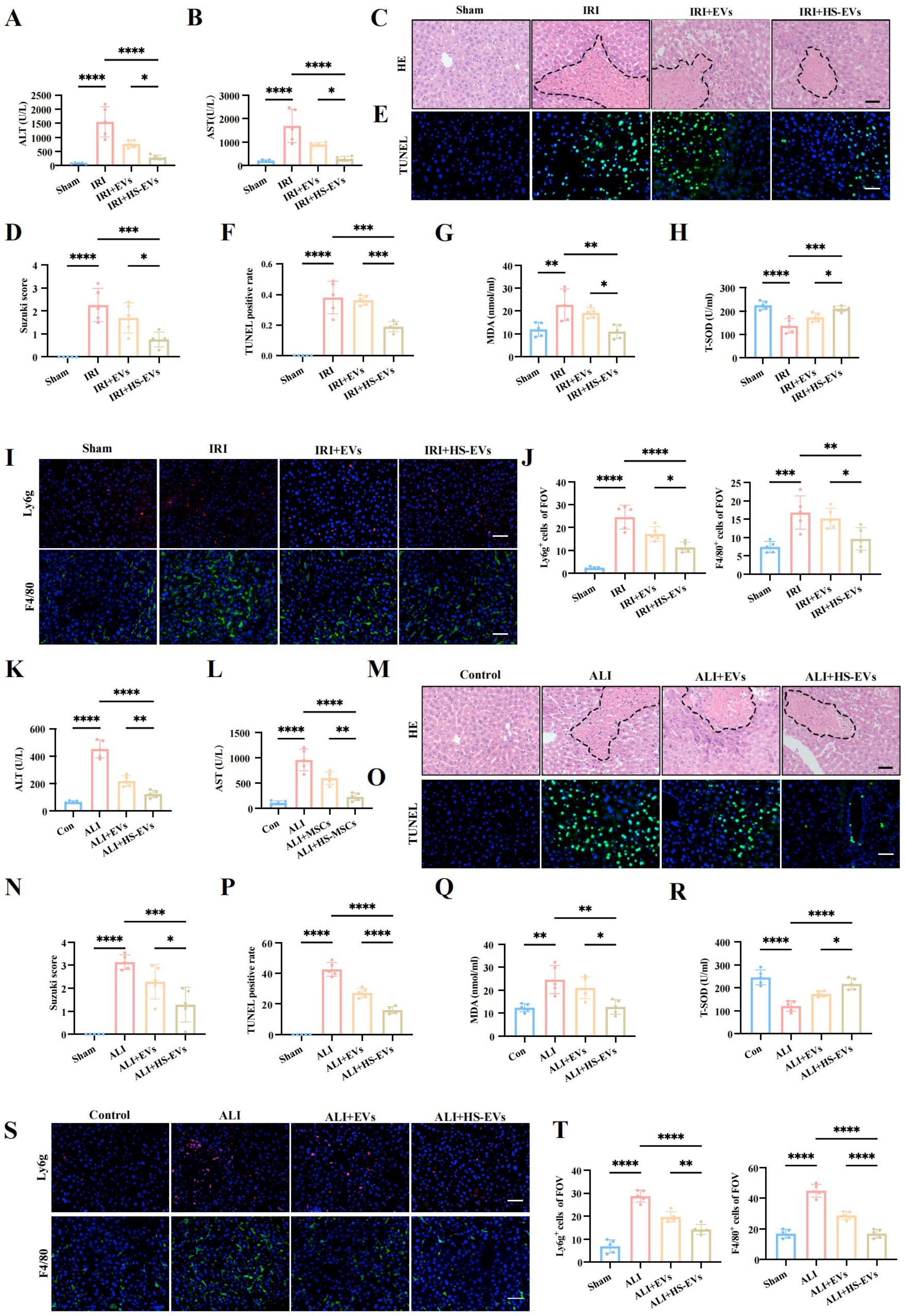

Figure S12. HS Treatment Heightened the Hepatoprotective potentials of MSC-EVs in the female liver injury models.

(A-B) Serum ALT and AST in Sham, IRI, IRI+MSCs, IRI+EVs and IRI+HS-EVs. (C-D) Representative images of H&E staining (the dotted line indicate areas of necrosis) on liver sections from different groups (Scale bar: 50 μm); The severity of liver injury was evaluated from histological section and scored according to Suzuki's injury criteria. (E-F) Representative images of TUNEL staining on liver sections from different groups (Scale bar: 50 μm); Statistical analyses of the percent of TUNEL-positive cells in each group. (G-H) Serum MDA and T-SOD in each group. (I-J) Representative images of Ly6G and F4/80 staining on liver sections from different groups (Scale bar: 50 μm); Statistical analyses of the percent of Ly6G-positive or F4/80-positive cells in each group. (K-L) Serum ALT and AST in Control, ALI, ALI+MSCs, ALI+EVs and ALI+HS-EVs. (M-N) Representative images of H&E staining (the dotted line indicate areas of necrosis) on liver sections from different groups (Scale bar: 50 μm); The severity of liver injury was evaluated from histological section and scored according to Suzuki's injury criteria. (O-P) Representative images of TUNEL staining on liver sections from different groups (Scale bar: 50 μm); Statistical analyses of the percent of TUNEL-positive cells in each group. (Q-R) Serum MDA and T-SOD in each group. (S-T) Representative images of Ly6G and F4/80 staining on liver sections from different groups (Scale bar: 50 μm); Statistical analyses of the percent of Ly6G-positive or F4/80-positive cells in each group. (n=5) *$p < 0.05$, **$p< 0.01$, ***$p< 0.001$, ****$p< 0.001$.

Fig. S13

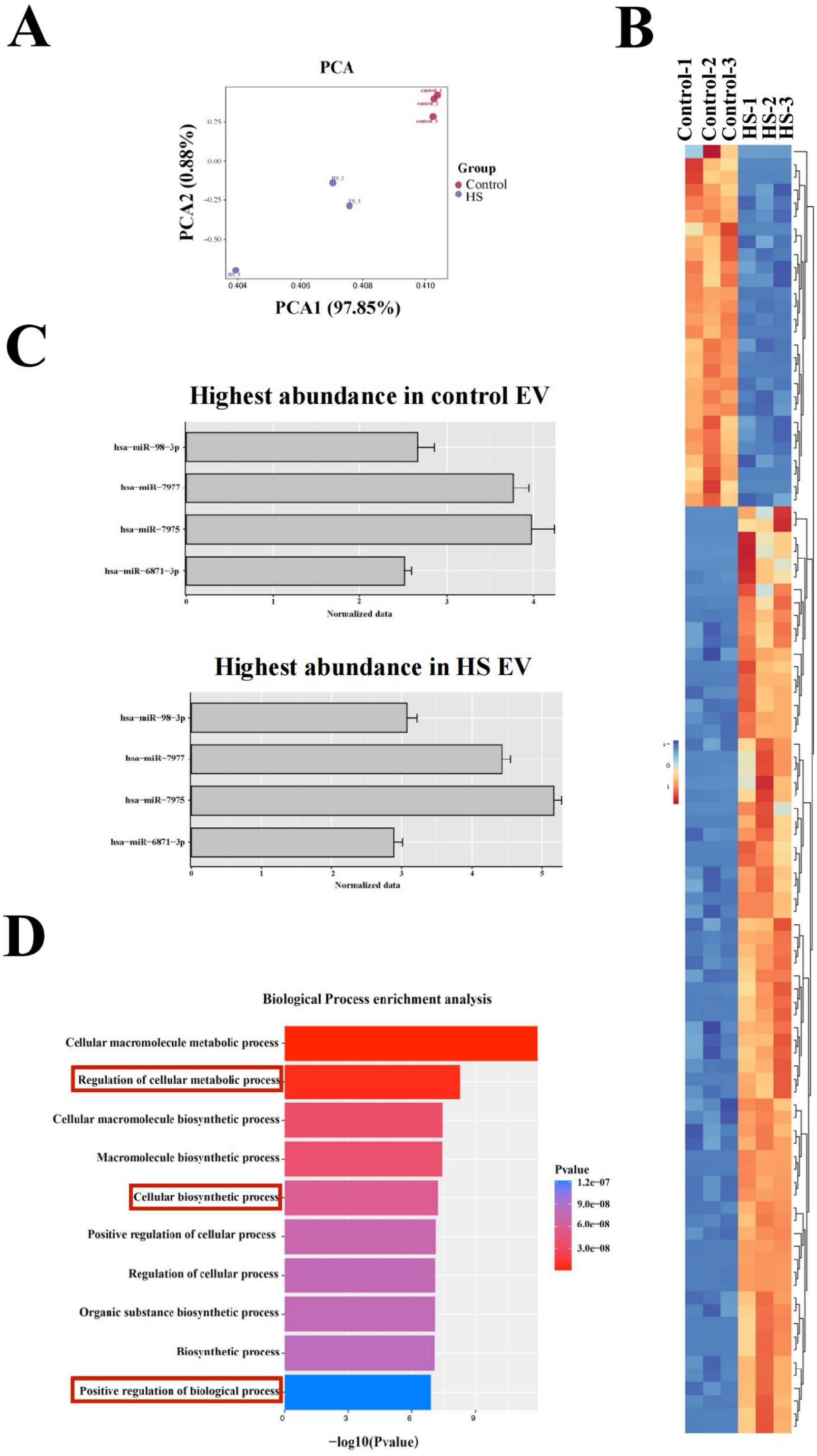

Figure S13. Distinct proteins and non-coding RNAs (ncRNAs) profiles in EVs from MSCs and HS-treated MSCs.

(A) PCA showed both intragroup repeatability and intergroup variability. (B) Heatmap showing differentially expressed proteins in the indicated groups. (C) The top 4 most abundant miRNAs in control and HS treated MSCs. (D) Enriched biological process related to the differentially expressed miRNA.

Supplementary Table 1: Reagents and Resources

Regent or Resource		
**Antibodies**	**Resource**	**Identifier**
Mouse anti-β-Actin	CST	Cat: 3700
Rabbit anti-CD63	abcam	Cat: ab271282
Rabbit anti-ALIX	CST	Cat: 2171S
Rabbit anti-TSG101	abcam	Cat: ab125011
Mouse anti-Rab27a	abcam	Cat: ab55667
Rabbit anti-GRP94	CST	Cat: 20292S
Rabbit anti-Beclin-1	abcam	Cat: ab62557
Rabbit anti-ATG5	CST	Cat: 12994
Rabbit anti-LC3B	CST	Cat: 3868S
Rabbit anti-TRPV2	abcam	Cat: ab272862
Rabbit anti-HA	CST	Cat: 3724S
Rabbit anti--Succinyllysine	PTM	Cat: PTM-401
Rabbit anti-ULK1	BioArt	Cat: ET1704-63
Rabbit anti-YWHAZ	CST	Cat: 7413
Rabbit anti-SUCLG2	Proteintech	Cat: 14240-1-AP
Mouse anti-OXCT1	Proteintech	Cat: 12175-1-AP
Rabbit Anti-KAT2A	Abcam	Cat:ab217876
Mouse anti-FLAG	CST	Cat: 14793S
Rabbit Anti-Sirt5	Abcam	Cat:ab259967
Rabbit-Anti SUCLA2	Abcam	Cat:ab202582
Mouse anti-UB	CST	Cat: 3936T
Rabbit anti-Lactyl Lysine	PTM	Cat: PTM-1401RM
Rabbit anti-GAPDH	CST	Cat: 2118
Mouse anti-Anti-Acetyllysine	PTM	Cat: PTM-105
Mouse anti-Malonylation	PTM	Cat: PTM-902
Rabbit anti-β -Hydroxybutylation	PTM	Cat: PTM-1201RM
Rabbit anti-Sumolyatiion-1	CST	Cat: 4930S
Rabbit anti-Sumolyatiion-2/3	CST	Cat: 4971S
Actin-Tracker Red-594	Beyotine	Cat: C2205S

FITC Conjugated Goat anti-rabbit IgG(H+L)	Beyotine	Cat: A0562
anti-human CD73-PE	BioLegend	Cat:344003
anti-human CD90-PE	BioLegend	Cat: 328109
anti-human 105-PE	BioLegend	Cat: 323205
anti-human CD11b-FITC	BioLegend	Cat: 982614
anti-human CD19-FITC	BioLegend	Cat: 302206
anti-human CD34-FITC	BioLegend	Cat: 343503
anti-human CD45-FITC	BioLegend	Cat: 982316
anti-human HLA-DR-FITC	BioLegend	Cat: 327005
HRP-conjugated anti-mouse IgG	CST	Cat: 982306
HRP-conjugated anti-rabbit IgG	CST	Cat: 300911
**Transfection Reagents**		
JetPRIME	Polyplus	Cat: 101000046
**Other Reagents**		
HBAD-mRFP-GFP-LC3	HANBIO	Cat:HH20230609CJF-AD01
Antifade mounting medium with DAPI	KeyGEN	Cat: KGF0282
Fluo-4 calcium ion detection kit	Beyotime	Cat: S061S
Colorimetric Histone Acetyltransferase Activity Assay (HAT)	ScienCell	Cat: 8668
Pierce™ GST Protein Interaction Pull-Down Kit	Thermo	Cat:21516
One-step TUNEL apoptosis detection kit (green fluorescence)	Beyotime	Cat: C1088
Malondialdehyde (MDA) Test Kit	Jiancheng Technology	Cat: A003-1
Total superoxide dismutase (T-SOD) assay kit	Jiancheng Technology	Cat: A001-1
Recombinant Human LDHA protein (His tag)	Elabscience	Cat: PDEH100141
YWHAZ Fusion Protein	Proteintech	Cat: Ag6654
OXCT1 Protein, Human, Recombinant (His)	TargetMol	Cat: TMPH-02151
Recombinant Human Succinyl-CoA ligase [GDP-forming] subunit beta, mitochondrial	CUSABIO	Cat: CSB-EP846636

(SUCLG2), partial In Stock		HU
Succinyl coenzyme A sodium salt	Sigma	Cat: S1129
Anti-FLAG® M2 Magnetic Beads	Sigma	Cat: M8823
EZview™ Red Anti-HA Affinity Gel	Sigma	Cat: E6779
**Plasmids**		
PCDNA3.1	MIAOLING BIOLOGY	Cat:
pCMV-SIRT5(human)-3xFLAG-Neo	MIAOLING BIOLOGY	Cat: P44526
pLV3-CMV-SUCLG2(human)-2-3 × FLAG-CopGFP-Puro	MIAOLING BIOLOGY	Cat:P50931
pCMV-SUCLA2(human)-3 × FLAG-Neo	MIAOLING BIOLOGY	Cat:P45443
pCMV-SDHA(human)-3×FLAG-Neo	MIAOLING BIOLOGY	Cat: P47875
pCDNA3.1-KAT2A(human)-FLAG	MIAOLING BIOLOGY	Cat:P20785
pLV3-CMV-YWHAZ(human)-3×HA-Puro	MIAOLING BIOLOGY	Cat:P57396
pLV3-CMV-YWHAZ(human)-L11R-3×HA-Puro	MIAOLING BIOLOGY	N/A
pCMV-OXCT1(human)-FLAG-SV40-Neo	MIAOLING BIOLOGY	Cat:P4044
pLV3-CMV-YWHAZ(human)-K11E-3×HA-Puro	MIAOLING BIOLOGY	Cat:N/A
**siRNA**		

NC siRNA F: 5’-TTCTCCGAACGAGTCACGT-3’	Genephar ma	N/A
NC siRNA R: 5’-ACGTGACTCGTTCGGAGAA-3’	Genephar ma	N/A
TRPV2-siRNA1 F: 5’- GGACAGAGGAAAGCUGGAUdTdT -3’	Genephar ma	N/A
TRPV2-siRNA1 R: 5’- AUCCAGCUUUCCUCUGUCCdAdT -3’	Genephar ma	N/A
TRPV2-siRNA2 F: 5’- CCAGUGCACAGAUGACUAUdTdT -3’	Genephar ma	N/A
TRPV2-siRNA2 R R: 5’- AUAGUCAUCUGUGCACUGGdAdT -3’	Genephar ma	N/A
SUCLG2 siRNA1 F: 5’-GGAGUGAGAGUUCAAAGAUTT-3’	Genephar ma	N/A
SUCLG2 siRNA1 R: 5’-AUCUUUGAACUCUCACUCCTT -3’	Genephar ma	N/A
SUCLG2siRNA2 F: 5’-CCAGGCUGCAGAUCAAAUUTT-3’	Genephar ma	N/A
SUCLG2siRNA2 R: 5’-AAUUUGAUCUGCAGCCUGGTT-3’	Genephar ma	N/A
OXCT1siRNA1 F: 5’-CAAGAAGGAGGAUCGCCCAUCAAAU-3’	Genephar ma	N/A
OXCT1siRNA1 R: 5’-AUUUGAUGGGCGAUCCUCCUUCUUG-3’	Genephar ma	N/A
OXCT1siRNA2 F: 5’-CAGAAGACAUCCAUAUUCCUCAGAU-3’	Genephar ma	N/A
OXCT1siRNA2 R: 5’-AUCUGAGGAAUAUGGAUGUCUUCUG-3’	Genephar ma	N/A
**Primers for RT–qPCR**		
GAPDH-F: 5’- CTGGGCTACACTGAGCACC-3’	Generay	N/A
GAPDH-R: 5’- AAGTGGTCGTTGAGGGCAATG -3’	Generay	N/A
TRPV2-F: 5’- AGTCAACCTCAACTACCGAAAGG-3’	Generay	N/A
TRPV2-R: 5’- CCGCATTGAAGAGCCGATCT-3’	Generay	N/A
